# Insights Into Central Nervous System Glial Cell Formation and Function From Zebrafish

**DOI:** 10.3389/fcell.2021.754606

**Published:** 2021-11-29

**Authors:** Sarah A. Neely, David A. Lyons

**Affiliations:** Centre for Discovery Brain Sciences, University of Edinburgh, Edinburgh, United Kingdom

**Keywords:** zebrafish, radial glia, Müller glia, astrocyte, OPC, oligodendrocyte, microglia

## Abstract

The term glia describes a heterogenous collection of distinct cell types that make up a large proportion of our nervous system. Although once considered the glue of the nervous system, the study of glial cells has evolved significantly in recent years, with a large body of literature now highlighting their complex and diverse roles in development and throughout life. This progress is due, in part, to advances in animal models in which the molecular and cellular mechanisms of glial cell development and function as well as neuron-glial cell interactions can be directly studied *in vivo* in real time, in intact neural circuits. In this review we highlight the instrumental role that zebrafish have played as a vertebrate model system for the study of glial cells, and discuss how the experimental advantages of the zebrafish lend themselves to investigate glial cell interactions and diversity. We focus in particular on recent studies that have provided insight into the formation and function of the major glial cell types in the central nervous system in zebrafish.

## Introduction

### The Importance of Glia

Although originally characterised as bystanders to neuronal function by Virchow in 1846, constituting the “glue” of the nervous system (Somjen, 1988), the possible roles of glia in supporting efficient nervous system function have since been vastly explored. It is now well established that glial cells are important throughout life, with recent research highlighting their significant contributions to nearly all aspects of nervous system function (Barres, 2008; Allen and Lyons, 2018). Since their initial description over a century ago, glial cells have been investigated in many diverse model organisms, each providing distinct strengths, with many aspects of glial biology highly conserved across species from invertebrates to humans (Freeman and Doherty, 2006; Lyons and Talbot, 2015; Shaham, 2015; Zuchero and Barres, 2015; Jäkel and Dimou, 2017; Yildirim et al., 2019). Here, we focus on the contribution of the zebrafish as a model organism to investigate the development and function of the major glial cell types in the vertebrate central nervous system (CNS), from radial glial cells, Müller glial cells, astrocytes, oligodendrocyte progenitor cells (OPCs) and oligodendrocytes (OLs) through to microglia.

## The Advantages of Zebrafish to Study Glial Cells

The zebrafish is well suited to follow the highly dynamic activities of glial cells, their interactions with other cells, and the molecular mechanisms that control their formation and function throughout life. This is because of a number of distinct advantages of the zebrafish as a vertebrate model system [as reviewed by Lieschke and Currie (2010) and Stewart et al. (2014)]. The small size of adult zebrafish (a few cm long) and their relatively quick life cycle (embryo to sexually mature adult in circa 2–3 months) make zebrafish a cost-effective model organism to maintain in large numbers. Secondly, the external development of zebrafish allows access to embryos from the single-cell stage, including for microinjections, e.g., for early genetic manipulation (Driever and Stemple, 1994; Kawakami et al., 2004). Additionally, the larval zebrafish remains optically transparent for the first weeks of its life, which enables molecules, cells and tissues of interest to be non-invasively imaged within the animal (Swinburne et al., 2015; Bin and Lyons, 2016). Furthermore, despite an analogous generation time when compared to rodents, zebrafish embryos develop very rapidly compared to other models, growing from a fertilised egg to a freely swimming larval fish which displays complex behaviours within a few days of egg fertilisation (Orger and De Polavieja, 2017). This rapid development, coupled with the fact that female zebrafish can produce hundreds of eggs at a time, facilitate large-scale screens on thousands of embryos and larvae over days to weeks. Also, and very importantly, zebrafish have a well conserved genome with other vertebrates (Howe et al., 2013). However, unlike other vertebrates, zebrafish have many duplicated genes following an additional whole genome duplication event in their ancestry (Howe et al., 2013). Despite this, zebrafish exhibit many conserved molecular mechanisms underpinning cellular physiology through to animal behaviour, including regulation of glial cell development (Jadhav et al., 2009; Goldman, 2014; Lyons and Talbot, 2015; Mathews and Appel, 2016b; D’Rozario et al., 2017; Jurisch-Yaksi et al., 2020). Together these properties allow the study of glial cells as they colonise the nervous system of the zebrafish in the first weeks of embryonic-larval life.

### Tools to Study Glial Cells and Their Cell-Cell Interactions *in vivo*

**FIGURE 1:**
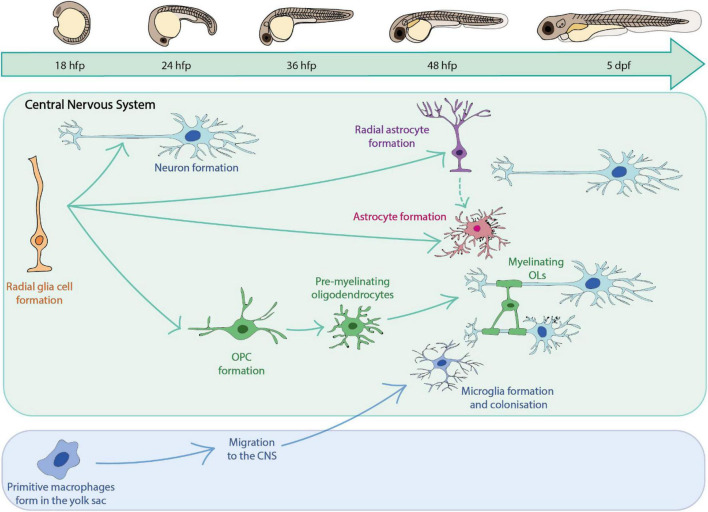
A summary of key glial cells of the zebrafish central nervous system and their timeline of development.

A major goal in the field of glial cell research is to understand the cellular behaviour and complex interactions of glia with other cell types, and how these combine to affect overall circuit formation and function. Zebrafish provide the opportunity to directly observe glial cell dynamics and cell-cell interactions *in vivo* over time in the living organism without the need for invasive surgical techniques, which are typically required for analogous analyses in other systems such as murine models (Hill et al., 2018; Hughes et al., 2018). High resolution imaging of fluorescently labelled glial cells of interest can be carried out in the optically transparent zebrafish larvae over the first 1–2 weeks of life, as glial cells populate the CNS (Lyons and Talbot, 2015; Preston et al., 2019; Figure 1). At yet later stages, live *in vivo* imaging can be carried out using zebrafish with mutations in pigment genes such as *golden*, *nacre* and *casper* (Lister et al., 1999; Lamason et al., 2005; White et al., 2008).

Advances in the development of fluorescent reporters means that morphological and functional characteristics of glial cells can be examined within the zebrafish [exemplified throughout and reviewed in Bin and Lyons (2016), Czopka (2016), and D’Rozario et al. (2017)]. These enable intricate processes such as OPC migration and proliferation (Kirby et al., 2006), myelin sheath formation and growth (Klingseisen et al., 2019), and the dynamic behaviour of microglia (Casano et al., 2016), to be investigated at high resolution in real time *in vivo*. In addition, reporters of cell state and function have become available, e.g., those with pH and ion sensitivity, which have enabled investigation of glial cell activity including Ca2+ activity in astrocytes (Chen et al., 2020), OPCs (Marisca et al., 2020), and myelinating oligodendrocytes (Baraban et al., 2018; Krasnow et al., 2018), the phagocytic properties of microglia (Peri and Nüsslein-Volhard, 2008; Hughes and Appel, 2020), and even functional activity of entire populations of neurons (Kim et al., 2017; Vanwalleghem et al., 2020) and glia (Baraban et al., 2018; Mu et al., 2019), including as they interact (Diaz Verdugo et al., 2019). In addition, various photo-activatable reagents are available and can allow, for example precisely timed glial cell ablation (Auer et al., 2018), or stimulation of migration (Sieger et al., 2012; Piller et al., 2021). Furthermore, integration of microscopy and behavioural platforms now enables in depth characterisation of glial cells during specific behaviours. For example, arenas wherein zebrafish are presented with virtual reality visual stimuli can now be used to measure neuronal and glial cell activity using optical methods that span from single cell resolution, through to brain-wide analyses of entire cell populations (Engert, 2012; Ahrens et al., 2013; Cong et al., 2017), providing invaluable insight into neural-glia control of circuit function in a live animal (Diaz Verdugo et al., 2019).

### Genetic Screening and Investigation

Another major goal in glial cell research is to identify genes required for their development, function and cell-cell interactions. One of the principal ways this has been investigated in the zebrafish is through the use of forward genetic screens, which provides a means for unbiased discovery of gene function. Briefly, adult animals (males in zebrafish) are exposed to mutagens to create random heritable mutations in the genome, with later generation offspring screened for phenotypes of interest, followed by identification of the causative lesion to link phenotype and genotype (Patton and Zon, 2001; Lyons and Kegel, 2019). Although initial forward genetic screens were carried out in invertebrate model organisms such as *Caenorhabditis elegans* and *Drosophila melanogaster* (Nüsslein-Volhard and Wieschaus, 1980; Brenner, 2003), innovative work by Streisinger et al. (1981) in the 1980s demonstrated that forward genetic strategies were also feasible in the zebrafish. In the 1990s large-scale mutagenesis-based forward genetic screens were carried out by the Nüsslein–Volhard and Driever laboratories, and highlighted the power of the zebrafish as a model for gene discovery in vertebrates (Driever et al., 1996; Haffter et al., 1996). Since these pioneering screens, which identified hundreds of mutations disrupting numerous biological processes, several dedicated forward genetic screens have been carried out to investigate genes essential for glial cells, including those regulating OPC, oligodendrocyte and microglial cell formation and function (Kazakova et al., 2006; Pogoda et al., 2006; Peri and Nüsslein-Volhard, 2008; Mathews et al., 2014; Mazaheri et al., 2014; Meireles et al., 2014; Shiau et al., 2014; Kearns et al., 2015; Shen et al., 2016; Villani et al., 2019). We will highlight recent insights throughout this piece and, for further information, we direct readers to the following reviews (Lawson and Wolfe, 2011; Kegel et al., 2019). In the now classic three generation forward genetic screen, following mutagenesis of male zebrafish, animals are crossed with female zebrafish to generate a cohort of first generation individuals carrying unique random mutations. These animals are used to seed a second generation, which nowadays often involves combining with transgenic reporters with fluorescently labelled cells or structures of interest to allow for facile screening of third generation offspring for disrupted phenotypes (Lyons and Kegel, 2019). Given the genetic tractability of zebrafish, it is now possible to select from many available stable transgenic lines which specifically label glial cells [e.g., Kirby et al. (2006), Jung et al. (2010), Preston et al. (2019), Chen et al. (2020), Marisca et al. (2020), Wu et al. (2020)]. Furthermore, mutations in potential glial cell-regulating genes can be investigated in the context of the entire circuit by studying effects on complex forms of neuronal activity or behaviours such as specific swimming patterns, or sensorimotor behaviours including escape responses or prey capture, which are displayed in zebrafish within just a few days of egg fertilisation (Bianco et al., 2011, 2015; Mcclenahan et al., 2012). Developments in genome and RNA sequencing now enable more rapid and accurate identification of the causative lesions that underlie phenotypes than following early screens (Bowen et al., 2012; Henke et al., 2013).

Alongside forward genetic screens, the use of reverse genetic approaches to interrogate specific genes of interest have provided insight into glia in zebrafish, presenting a complement to mutagenesis-based screening approaches. Unlike forward genetic screens, wherein random mutations are induced in the genome, reverse genetic approaches utilise the genetic tractability of the zebrafish to apply targeted approaches to investigate specific gene function. One of the most popular techniques initially used to interrogate gene function in zebrafish utilised synthetic morpholino antisense oligonucleotides (MOs), which either block mRNA splicing or protein translation (Nasevicius and Ekker, 2000). These tools provided fundamental insights into the development of glial cells in zebrafish, particularly with respect to investigating the conservation in zebrafish of genes known to regulate glial cell development in mammals e.g., Park et al. (2002, 2004). However, although MOs provide a straightforward means to inhibit gene function, they have a number of limitations including off-target effects, variable gene knockdown and depleted efficiency over time, meaning great care must be taken in their use (Stainier et al., 2017). As for essentially all model organisms, the employment of recently discovered gene targeting approaches have revolutionised genetic analyses of zebrafish. For example, genetic knock out or knock in zebrafish can now be efficiently generated using Zinc finger nuclease (ZFN) technology, transcription activator-like effector nucleases (TALENS), and most recently, and most extensively, CRISPR/Cas9-based strategies [for reviews see Huang et al. (2012), Gaj et al. (2013), Ma and Liu (2015), Liu et al. (2017, 2019), Cornet et al. (2018), and Hans et al. (2021)].

The efficiency of new reverse genetic technology is continually improving, enabling more rapid investigation of genes for glial function. One example of this is the use of CRISPR/Cas9 strategies that can now be utilised to reliably induce somatic mutations with high efficiency in F0 embryos, called “crispants.” These significantly increase the speed with which genetic manipulations can be investigated (Keatinge et al., 2021; Klatt Shaw and Mokalled, 2021; Kroll et al., 2021), which can allow the investigation of duplicated genes, those with potentially redundant functions and even multiple pathways in parallel. From a practical view, these technologies reduce the personnel and space requirements of mutagenesis-based forward genetic screens, thereby allowing scalable investigation of gene function by most laboratories. Despite the efficiency of constitutive gene targeting in zebrafish, cell-type specific analyses of gene function is more technically challenging. A long-standing method to assess cell autonomous vs. non-autonomous gene function in zebrafish has been the creation of genetic chimeras by cell transplantation, which has been used to assess the roles of various factors in zebrafish glia, but is a method most suited to the analysis of single cell behaviour, given its chimeric nature (Lyons et al., 2009; Monk et al., 2009; Perlin et al., 2011; Mensch et al., 2015). Another approach widely used in the field is the expression of specific genes under the control of cell type specific drivers, e.g., the expression of wild-type genes on a mutant background, or the expression of dominant negative or constitutively active forms of genetic regulators, which has also provided insight into glial function in zebrafish (Supplementary Table 1). More recently, the creation of cell type specific knockout zebrafish using CRISPR-based strategies has also been carried out (Ablain et al., 2015), and strategies to do so are accumulating and show great promise. Although additional work remains to optimise such approaches for widespread analysis of candidate function in a cell type specific manner, they have already been utilised to provide insight into glial cell specific gene function in zebrafish (Chen et al., 2020; Marshall-Phelps et al., 2020). Complementary molecular approaches to profile cells including RNA sequencing, which is well established in zebrafish (Mazzolini et al., 2019; Wheeler et al., 2019; Farnsworth et al., 2020; Marisca et al., 2020) and, more recently, spatial transcriptomics, which is being optimised for use in zebrafish (Holler et al., 2021; Okochi et al., 2021), provide invaluable platforms for future investigation of the molecular basis of glial cell function in the zebrafish.

In parallel to forward and reverse genetic approaches to assess gene function, the field of optogenetics has revolutionised neuroscience by allowing researchers to take control of the functional firing and signalling properties of neurons and glial cells (Warden et al., 2012; Cho et al., 2016; Kato et al., 2019; Mu et al., 2019; Lee et al., 2020). A principal set of tools in optogenetics are light-sensitive ion channels, which can be activated to control the electrical activity of cells. Given the ease of transgenesis, small size and optical clarity of zebrafish embryos and larvae, it is no surprise that numerous optogenetic actuators have been employed in zebrafish, principally to assess neuronal circuit function (Antinucci et al., 2020). Furthermore, the emergence of various methods to influence signalling pathways in a light-inducible manner (Arrenberg et al., 2009; Buckley et al., 2016; Harris et al., 2020; Mruk et al., 2020) with readouts of many aspects of cellular function, provides exciting opportunities for future study of glial cells, and the importance of neuron-glia interactions in circuit function in zebrafish.

### Chemical Screening

As well as their advantages for genetic screening, zebrafish also provide a platform for drug discovery in a vertebrate model. Their rapid external development, small size (head to tail length of larvae with all major glial cell types present circa 5 mm long), and ability to absorb compounds through their skin, allows simple drug administration to live animals, enabling testing of thousands of compounds, even in multi-well plate format (Macrae and Peterson, 2015). Furthermore, the use of zebrafish enables multiple readouts of drug effects. These include simple phenotypic assessment of general health, development, organ growth and cardiovascular function. Additionally, behavioural readouts can be used to determine drug toxicity, alongside specific transgenic reporters for more in-depth cellular analysis. Since their first use in zebrafish, chemical screens have identified many promising compounds for various biological processes, with several taken to clinical trials [reviewed in Patton et al. (2021) and references therein]. In the context of glial biology, chemical screening based approaches have been utilised to identify compounds to enhance oligodendrogenesis and myelination (Buckley et al., 2010; Early et al., 2018), regulators of astrocytic function (Wheeler et al., 2019) and glial regulators of nerve regeneration (Bremer et al., 2017). Indeed, the investigation of glial cells in the context of regeneration and disease is now intensely investigated across models (Hamon et al., 2016; Cayre et al., 2021) and is a burgeoning aspect of research (Franklin et al., 2020; García-García et al., 2020; Eastlake et al., 2021; Hammond et al., 2021; Schirmer et al., 2021) that will form the basis of future reviews. In this piece, however, we will focus on the larger body of work that to date has investigated the development of glial cells in zebrafish, and begun to investigate their fundamental functional roles in the healthy nervous system.

## Glial Cells in the Zebrafish; Origins, Differentiation and Function

Studies in zebrafish have provided insights into glial cells of the CNS, peripheral nervous system (PNS) and indeed those that lie at and play essential roles at the boundary of the CNS and PNS. Insights into how zebrafish have informed our understanding of peripheral and boundary glia have been reviewed elsewhere (Kucenas, 2015; Monk et al., 2015; Ackerman and Monk, 2016; D’Rozario et al., 2017; Fontenas and Kucenas, 2018; Muppirala et al., 2020) and so we will focus this review on the major glial cells of the CNS summarised in Figure 1.

### Glia of the Zebrafish Central Nervous System

#### Radial Glial Cells

**FIGURE 2:**
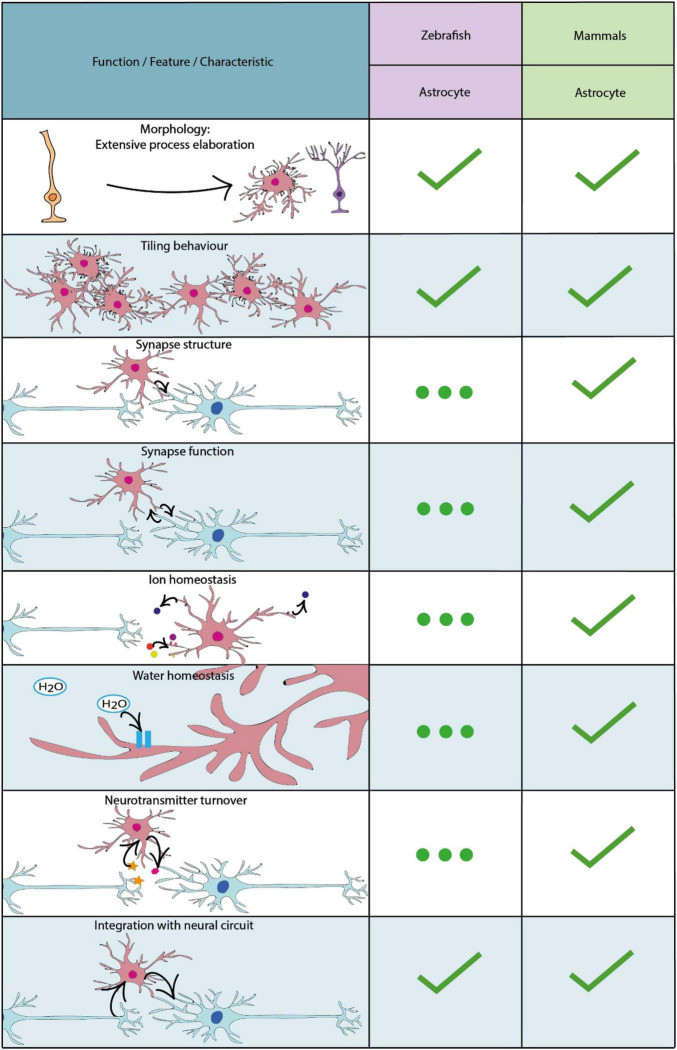
Key characteristics and functional properties of zebrafish and mammalian astrocytes. For simplicity we refer to astrocytes as cells with a radial morphology, or those which have adapted a morphology akin to radial astrocytes. The use of “…” refers to areas of astrocyte biology which require further investigation.

The first cells in the zebrafish to emerge with glial characteristics are radial glial cells which develop with the formation of the neuroepithelium from approximately 10 hours post fertilisation (Kim et al., 2008). Like other vertebrates, radial glial cells in the zebrafish are classified by their characteristic radial processes, which span from the basal to the apical (ventricular) surfaces of the neuroepithelium. Indeed, radial cells with glial characteristics represent the major progenitor cell of the early developing CNS, which can both self-renew and give rise to differentiated cell types, first to neurons, and later to oligodendrocyte progenitor cells, and then to astrocytes (Lyons and Talbot, 2015; Jurisch-yaksi, 2020). In mammals, following developmental neurogenesis and throughout the majority of the neuraxis, most radial glial cells ultimately detach from the ventricular zone, losing their radial morphology and subsequently contributing to the generation of bona fide astrocytes (Kriegstein and Götz, 2003). In other regions of the mammalian CNS, radial glia adopt heterogeneous states with various potentials to contribute to new cell generation throughout life (Götz et al., 2015; Falk and Götz, 2017). In the zebrafish however, neurogenesis is much more protracted and can, in principle, occur throughout the majority of the neuraxis throughout life (Kroehne et al., 2011; Kyritsis et al., 2012; Jurisch-Yaksi et al., 2020). Mirroring this capacity to serve as progenitor cells throughout life, a large proportion of radial glia in zebrafish retain their radial morphology life-long. In addition some radial glia appear to transition into a state with more specialised functions analogous to mammalian astrocytes. For example, both zebrafish radial glial cells and mammalian astrocytes share expression of the water transport protein aquaporin-4 and glutamate transporter Eaat2b, suggesting that they may conduct similar functions including regulating CNS water homeostasis and levels of neurotransmitters in the extracellular space (Grupp et al., 2010; McKeown et al., 2012; Papadopoulos and Verkman, 2013). Indeed, more recent studies have shown that some radial glial-like cells adopt gross morphological and functional features that overlap with those of mammalian astrocytes (Mu et al., 2019), and yet others appear to differentiate into bona fide astrocytes as discussed in the “Radial Astrocytes” and “Astrocytes” sections and summarised in Figure 2 below.

#### Müller Glial Cells

In the mammalian CNS, additional glial cells with radial morphologies are known to be present in specific brain areas, e.g., Bergmann glia in the cerebellum and Müller glia in the retina, the latter of which have been extensively investigated in zebrafish. The vertebrate retina consists of six principal neuronal cell types and one primary glial cell, the Müller glial cell. Müller glial cells have a radial morphology (akin to radial glia) and extend over all three retinal layers, giving them the ability to contact neurons, maintain homeostasis and influence retinal structure (Goldman, 2014). Several developmental regulators which control the highly complex radial morphology of Müller glia cells and their even spacing throughout retinal layers have been identified in the zebrafish by utilising CRISPR based screening (Charlton-Perkins et al., 2019). Interestingly, a number of these appear to be highly conserved across species (Fairchild et al., 2018), suggesting that Müller glia morphogenesis is regulated by highly conserved differentiation programmes. Genes including *pax2a*, *itga5*, *itga6* and *itgb1a*, as well as those in the *nephrins* and *cadm* gene families have been prioritised as interesting candidates for further future investigation (Charlton-Perkins et al., 2019).

Under normal conditions Müller glial cells have several conserved functions across model organisms, including recycling neurotransmitters, maintaining ionic balance and interacting with retinal microglial cells. For example, mammalian Müller glia, like their radial glial counterparts, express glutamate transporter EAAT2, and expression of Eaat2 orthologs is also observed in zebrafish Müller glia (Niklaus et al., 2017), which mediates removal of excess glutamate released from photoreceptor synapses, implying conservation in the roles of these cells across vertebrates. Indeed, when Eaat2a was depleted in zebrafish larvae, reduced electroretinographic responses were recorded (Niklaus et al., 2017), supporting the conclusion that Müller glia can influence synaptic function in the zebrafish, as in mammals. Furthermore, Müller glia have been shown to play an essential role in maintaining the tissue integrity of the retina, as retinas in zebrafish lacking Müller glia cells tear apart due to reduced tensile strength (MacDonald et al., 2015).

Similar to radial glial cells, Müller glia have been shown to be highly regenerative in response to injury in zebrafish as Müller glia cells can regenerate neurons, as well as all major retinal cell types (Powell et al., 2016; Sifuentes et al., 2016; Wan and Goldman, 2016). In contrast, Müller-glia derived progenitors in mammalian models have a more limited capacity to regenerate different cell types (Goldman, 2014). Therefore, future studies investigating the differences between mammalian and zebrafish Müller glia cells will be essential to determine the signalling pathways that are essential for efficient retinal repair.

The range of roles carried out by Müller glia cells in the retina highlights the functional diversity of glial cells with radial morphologies in different regions of the CNS. In the zebrafish, many distinct markers have been used to identify different radial glial cell populations (Bernardos and Raymond, 2006; Cuoghi and Mola, 2009; Farnsworth et al., 2020), with some cell morphologies and functions displaying characteristic biology assigned to radial glial cells whilst others are more reminiscent of astrocyte-like cells.

#### Radial Astrocytes

Studies in mammals have made it entirely clear that astrocytes are potent regulators of various aspects of neural circuit formation and function (Freeman, 2010; Clarke and Barres, 2013; Stogsdill and Eroglu, 2017; Dallérac et al., 2018; Farhy-Tselnicker and Allen, 2018; Perez-Catalan et al., 2021; Sancho et al., 2021). Astrocytes play a number of essential roles in the mammalian nervous system including modulating synapse formation, pruning, and physiology (Chung et al., 2015; Farhy-Tselnicker and Allen, 2018). Additionally, astrocytes can metabolically support the CNS, with extensive interactions with the vasculature and most major cell types of the CNS, including neurons, OPCs, oligodendrocytes and microglia (Clemente et al., 2013; Chung et al., 2015; Allen and Eroglu, 2017; Nutma et al., 2020). Reflecting their multiple roles, mammalian astrocytes have a complex morphology with an array of cellular processes that make connections with a host of these cellular targets (Allen and Lyons, 2018). However, the extent to which astrocyte-like cells with such complex morphology exist in zebrafish, or contribute to neural circuit structure and function was, until recently, quite unclear. In a tour de force study, Mu et al. (2019) showed that radial glial/astrocyte-like cells with complex morphologies do indeed exist in zebrafish and play an important role in neural circuit computations. In this study cells with both a radial process and an endfoot at the ventricular surface also had a huge network of cellular processes in synaptic regions. By whole-brain *in vivo* imaging of calcium transients of these cells as well as neurons while zebrafish were executing a specific behaviour, the authors were able to ascribe a role for these radial astrocytes in circuit computations (Mu et al., 2019). In this study, Mu and colleagues investigated the mechanisms of futility-induced passivity, or giving-up behaviour, which is a behaviour employed by many animals to conserve energy in-between high-activity phases (Warden et al., 2012; Andalman et al., 2019). Zebrafish elicit a visuomotor behaviour called the optomotor response to stabilise their position in the moving currents of river water, e.g., to not be swept downstream in a river upon an increasing current (Orger et al., 2000; Vladimirov et al., 2014). This response can be recapitulated experimentally in a virtual reality system by projecting moving bar patterns to the fish to simulate water flow and, in response, zebrafish elicit forward swims to stabilise their position relative to the moving bars. In one iteration of this experimental paradigm, animals are actually head-restrained using agarose while the bar patterns are projected and fictive swimming is induced, which can be assessed by electrophysiological recording of motor output. Sensory feedback corresponding to this measured fictive motor output is then provided back to the animal by way of adjusting the moving bar pattern. However, the experimenter can change the sensory feedback by altering the moving bar patterns. For example, if one simply keeps the bars moving at the same speed, i.e., not adjusted in line with the fish’s predicted motor output, the fish will have the “experience” of being swept along by the virtual current, despite its attempts to stabilise its position. After a certain period (tens of seconds) with such feedback, animals “give-up” on this apparently futile behaviour, hence the term futility-induced passivity.

By carrying out whole-brain light-sheet imaging of calcium activity in effectively all brain neurons, together with that of radial astrocytes during futility-induced passivity, the authors showed that while the activity of certain specific neurons decreased upon the switch to giving up, radial astrocyte activity in specific brain regions increased, and in fact proceeded, both the decrease in neuronal activity and the switch in behaviour. This implicated radial astrocytes in driving the giving up behaviour. The causal role of radial astrocytes in regulating both neuronal activity and the switch to the passive state was investigated in numerous complementary manners, using optogenetic, chemogenetic, pharmacological and cell-ablation based manipulations of radial astrocytes (Mu et al., 2019). Although the precise circuit mechanisms remain to be fully defined, this study provides direct evidence that radial astrocytes can integrate and in turn regulate neuronal activity and behaviour in a living vertebrate. Even though the cells whose activity was imaged in this study were referred to as radial astrocytes it remained unclear what proportion of these astrocyte-like cells actually retained a radial-like morphology. Given the historical lack of documentation of cells with a truly astrocytic (star-like) non-radial morphology in anamniotes, it was assumed that essentially all such cells may retain a radial morphology and thus a hybrid radial glial/astrocytic state. However, a very recent study has shown that this is not necessarily the case and that cells with definitive astrocyte-properties exist in fish (Chen et al., 2020) (Figure 2).

#### Astrocytes

It is important to note that the study of glial cells in zebrafish remains of relatively modest volume compared with that of mammalian models, and until the advent of transgenic reporter tools, approaches to visualise glial cell morphology in zebrafish typically relied on a restricted number of antibody-based labelling approaches (Grupp et al., 2010). Therefore, the fact that cells with a bona fide astrocyte nature had not been identified reflected more absence of evidence than evidence of absence. Indeed, it was a breakthrough in transgenic reporter technology that unlocked the evidence for astrocytes in zebrafish. Starting with an *in situ* hybridisation screen of candidate astrocyte markers from the mammalian literature, Chen et al. (2020) selected the glutamate aspartate transporter (Glast) as a strong candidate maker of astrocytes in zebrafish. Upon making transgenic constructs and stable reporter lines using *glast* regulatory sequence, Glast expressing cells were shown to have a number of similarities to mammalian astrocytes (Figure 2). Using live *in vivo* imaging Chen and colleagues first demonstrated that zebrafish Glast-expressing cells transform in morphology from radial glial cells into cells that display complex morphologies akin to mammalian astrocytes (Figure 2). Furthermore, Glast-expressing cells display tiling behaviours, in order to maximise CNS coverage, similar to mammalian astrocytes (Bushong et al., 2002) (Figure 2). Using the *glast* regulatory sequence to drive expression of a genetically encoded indicator defined patterns of Ca2+ activity highly characteristic of those seen in mammals (Nett et al., 2002; Wang et al., 2019) were observed (Figure 2), which importantly were shown to be sensitive to regulation by norepinephrine, as in mammals (Ding et al., 2013; Paukert et al., 2014). Additionally, Glast-expressing cells in the zebrafish expressed glutamine synthetase in their somata and processes, which is also enriched in mammalian astrocytes and is known to be essential for neuronal interactions (Norenberg and Martinez-Hernandez, 1979). Interestingly, astrocyte-like Glast-expressing cells in the zebrafish spinal cord were also found to closely associate with synapses, identified utilising the presynaptic vesicle glycoprotein 2A, suggesting that zebrafish astrocytes may play an important role in mediating synaptic formation and function (Figure 2). Given the large body of literature indicating that astrocytes regulate effectively all stages of synapse formation and function in mammals (Chung et al., 2015; Farhy-Tselnicker and Allen, 2018), it will be important to determine to what extent this is true in zebrafish (Figure 2). For example, given their close proximity to synapses, similar to astrocytes in mammalian models (Stogsdill et al., 2017), it is possible that zebrafish astrocytes may play a role in the clearance of ions and neurotransmitters from the extracellular space, and potentially also in gliotransmission. Given the power of zebrafish for investigation of cell structure, function and interactions at sub-cellular resolution, one can envision future studies that combine high resolution imaging of glial cells with functional imaging of neuronal and synaptic activity during the execution of specific behaviours, to explore the regulation of synaptic formation and function by astrocytes in zebrafish. One area of increasingly obvious importance from studies in mammals is that of heterogeneity with respect to subtypes and states of astrocytes (Oberheim et al., 2012; Poskanzer and Molofsky, 2018; Bellini et al., 2019), which will also be important to investigate in zebrafish by combining molecular profiling studies with the advantages of visualising cell structure and function *in vivo* using zebrafish in health, and ultimately also in disease-relevant experimental paradigms.

#### Oligodendrocyte Lineage Cells

##### Oligodendrocyte Progenitor Cells

**FIGURE 3:**
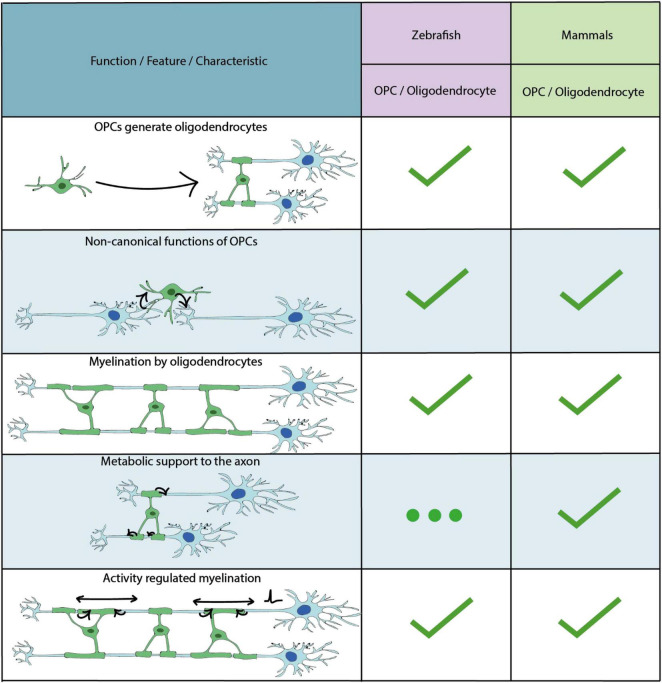
Key characteristics and functional properties of zebrafish and mammalian OPCs and oligodendrocytes. The use of “…” refers to areas of OPC/oligodendrocyte biology which require further investigation.

In addition to astrocytes and neurons, radial glial cell progenitors can also produce OPCs. OPCs (also known as NG2 cells) are an abundant group of progenitor cells in the CNS throughout life, which are principally known for their ability to generate myelinating oligodendrocytes (Mitew et al., 2014) (Figure 3). OPCs and oligodendrocytes are, to date, the most extensively studied glial cell lineage in zebrafish, with the focus having been on their formation and functions in the embryonic hindbrain and spinal cord. The majority of such early born OPCs in the zebrafish initially derive from the progenitor of motor neuron (pMN) domains of the ventral CNS, as in mammals (Lu et al., 2002; Park et al., 2002; Zhou and Anderson, 2002; Shin et al., 2003; Lamason et al., 2005; Li et al., 2007; Kucenas et al., 2008). Later in development, OPCs and oligodendrocytes in the zebrafish and other vertebrates colonise the entire brain (Richardson et al., 2006; Bergles and Richardson, 2016), but because these have been much less extensively studied in zebrafish we will focus on studies which have investigated the development of OPCs from the pMN domain.

###### Oligodendrocyte Progenitor Cell Specification

As in mammals, pMN progenitors in zebrafish initially generate motor neurons, then switch to producing OPCs in a process tightly regulated by a collection of transcription factors, signalling molecules and extracellular cues. For example, various transcription factors including Olig2 and Nkx2.2, and signalling pathways including Hedgehog have all been shown to have important conserved roles in OPC specification in zebrafish and mammals (Lu et al., 2000, 2002; Park et al., 2002; Kirby et al., 2006; Sun et al., 2006; Li et al., 2007; Kucenas et al., 2008; Lyons and Talbot, 2015) (Supplementary Table 1). More recently, mechanisms of Sonic hedgehog signalling regulation were investigated by Scott et al. (2020) who explored the effects of transcriptional repressor prdm8 on OPC specification. By live *in vivo* imaging they show that pMN cells in *prdm8* mutant embryos have elevated Sonic hedgehog signalling and prematurely switch from motor neuron to OPC production (Scott et al., 2020). Intriguingly, Prdm8 was also shown to influence the fate of oligodendrocyte lineage cells, as *prdm8* mutant embryos have increased numbers of oligodendrocytes but lack sufficient OPCs. Together, this suggests that the morphogen Sonic hedgehog plays an important role in controlling the timing of OPC specification in zebrafish (Ravanelli and Appel, 2015; Scott et al., 2020). Similarly, the Notch canonical signalling pathway also influences the timing of the pMN domain progenitor differentiation switch from motor neurons to OPCs. This has been shown in zebrafish where reduced Notch activity induced excess formation of primary motor neurons and reduced numbers of OPCs (Appel et al., 2001; Supplementary Table 1). In contrast, constitutive Notch activity promoted OPC production (Appel et al., 2001; Supplementary Table 1). Complementing this study, Snyder et al. (2012) found that disrupting *fbxw7*, a ubiquitin ligase that targets Notch for degradation, results in excess OPC production (Supplementary Table 1). Taken together, these data suggest that Notch signalling tightly regulates the timing of the pMN progenitor differentiation switch, and therefore plays a central role in OPC specification.

Concomitant with their specification, OPCs in zebrafish and mammals delaminate from the neuroepithelium and migrate towards their axonal targets. One interesting candidate which has been identified in zebrafish to affect the timing of neuroepithelial delamination to OPCs in the zebrafish is the microRNA mir-29, which can regulate the expression of the polarity genes *pard3* and *prkci* (Hudish et al., 2013; Supplementary Table 1). How OPCs are specified in other regions of the neuraxis and at later stages, will require ongoing analyses, and in particular the way in which radial-glial-like progenitors present in zebrafish can give rise to OPCs following injury or demyelination awaits in-depth analyses ahead of comparison with mammals.

###### Oligodendrocyte Progenitor Cell Proliferation and Migration

Following delamination from the neuroepithelium OPCs in mammals proliferate extensively and migrate widely to take up positions throughout the parenchyma in which they both continue to proliferate and also adopt specialised functions, including the generation of myelinating oligodendrocytes (Figure 3). In contrast, at the stages and regions principally studied to date in zebrafish, OPCs have a relatively small volume to colonise. Indeed, the total distance that OPCs migrate from the pMN domain in the ventral spinal cord to nearby ventral axonal tracts where oligodendrocytes first differentiate is on the order tens of microns with dorsal regions of the hindbrain or spinal cord remaining only 100–200 μm from the pMN domain. These small distances mean that the OPC population doesn’t undergo a huge degree of expansion through proliferation and migration at early embryonic stages. To what extent principles of OPC population expansion and migration observed in mammals, e.g., whereby OPCs follow the brain vasculature to migrate over long distances (Tsai et al., 2016), are conserved in fish will require analyses at later stages and in other regions of the CNS when and where migratory routes are longer and, likely more complex. Nonetheless, several cell-cell interactions, as well as specific molecular factors have been identified that influence both OPC proliferation and migration in the developing zebrafish (Supplementary Table 1).

For example, OPC proliferation appears to be responsive to the density of axons fated for myelination in the embryonic zebrafish CNS, with OPC proliferation reduced in a mutant that lacked axons fated for myelination in the posterior spinal cord, Almeida and Lyons (2016). One set of axonal signals known to regulate many aspects of the oligodendrocyte lineage in mammals, including OPC proliferation, are those associated with neuronal activity (Geraghty et al., 2019). Indeed, a recent study has indicated that neuronal activity also influences OPC proliferation in the zebrafish where a pharmacologically-induced increase in neural activity resulted in increased OPC proliferation (Marisca et al., 2020). Although the axonal signals (activity-related or not) that regulate OPC proliferation in zebrafish await identification, receptors on oligodendrocytes that influence proliferation have been uncovered (Supplementary Table 1). In addition to proliferation, neuronal activity has also been implicated in regulating OPC migration in mammals (Harlow et al., 2015) and recently it was found that disruption to the AMPA receptor subunit GluR4A (encoded by *gria4a*) expressed on OPCs impaired migration in zebrafish (Piller et al., 2021) (Supplementary Table 1).

Following migration to their target regions, OPCs undergo a set of cell-cell interactions to demarcate the space in which they will ultimately reside. During this tiling behaviour OPCs continuously extend and retract processes to sample their environment and appear to be highly influenced by contact with surrounding OPCs, whereby contact between distinct OPCs appears to induce repulsion of contacting processes (Kirby et al., 2006). Indeed, when a single or small group of OPCs are ablated, nearby OPCs increase cell division to replenish numbers, fill the space, and reinitiate tiling behaviours (Kirby et al., 2006). This appears to be a largely conserved mechanism between species, given that similar observations were made in the cortex of mature mice, where neighbouring OPC processes exhibited contact-mediated withdrawal, and increased proliferation following damage to neighbouring OPCs (Hughes et al., 2013). Recently, in a mouse model, the molecular mechanism for this OPC-OPC interaction was proposed (Chavali et al., 2020), conservation of which remains to be tested in zebrafish, but appears likely, given the remarkable similarity in associated cell behaviour across species.

###### Oligodendrocyte Progenitor Cell Diversity

The broadly accepted view of OPCs is that they are principally sources of myelinating oligodendrocytes. However, it is now becoming clear that OPCs can exhibit a diverse array of functions beyond their roles in making myelinating oligodendrocytes (Harrington et al., 2020; Akay et al., 2021; Clayton and Tesar, 2021) (Figure 3).

The existence of OPCs with potentially distinct fates was first investigated in zebrafish by Kucenas et al. (2008), who showed that OPCs that express the transcription factor Nkx2.2a, an ortholog of rodent Nkx2.2, typically differentiate into myelinating oligodendrocytes, whereas OPCs that do not express *nkx2.2a* mostly remain as non-myelinating OPCs. The fate of distinct zebrafish OPCs was recently investigated further in a study that combined fate mapping of individual OPC clones, morphological characterisation of their dynamic behaviour by time-lapse microscopy, and associated single cell RNA sequencing (Marisca et al., 2020). This study confirmed the existence of two discrete groups of OPCs in the zebrafish spinal cord, those with cell bodies located within axonal tracts and those with cell bodies surrounded by the cell bodies of other cells, principally neurons (Marisca et al., 2020). Interestingly, OPCs with cell bodies located in the axon-dense areas tended to directly generate myelinating oligodendrocytes, whereas those with cell bodies in neuron/soma-rich areas tended to self-renew and remain relatively stable, although these cells could also generate the OPCs that moved to axon-rich areas. These two OPC types displayed both unique branching morphologies and very different patterns of calcium activity and interactions with axons. Interestingly, and perhaps surprisingly, OPCs with their cell bodies within the neuron-rich areas had higher levels of calcium activity, but yet infrequently differentiated, despite having extensive processes that made very stable contact with axons. Interestingly and correspondingly, these OPCs had a molecular signature of being responsive to neuronal activity, and it was these cells whose proliferation was responsive to changes in activity noted earlier (Marisca et al., 2020). This suggests that although neuronal activity can regulate OPC proliferation, it may not, directly at least, influence oligodendrocyte differentiation.

The function of OPCs beyond their generation of oligodendrocytes has been hard to experimentally disentangle, but has recently been investigated in zebrafish, by taking advantage of the presence of abundant OPCs in a brain area, the optic tectum, that does not become myelinated in the developing larva. The optic tectum is the largest retino-recipient brain structure in zebrafish, receiving input from a large proportion of retinal ganglion cell axons and is analogous to the superior colliculus in mammals. Upon arrival at the tectum, distinct retinal ganglion cell axons enter specific tectal layers where they make precise connections with defined tectal neuron dendrites (Erskine and Herrera, 2007). To investigate a potential role for OPCs in connectivity with target neurons, Xiao et al. (2021) ablated tectal OPCs and found that this resulted in enlarged axonal arbors in the tectum and branching of retinal ganglion cell axons beyond the tectal neuropil where target dendrites are located. Indeed, live imaging implicated OPCs in regulating axonal pruning/remodelling *in vivo*, which had previously been predicted by *in vitro* studies in mammals (Goldberg et al., 2004). Very interestingly, ablation of tectal OPCs in zebrafish also resulted in impairments to visual function. Recent investigation in rodent models suggest that OPCs can engulf axons in mammals (Buchanan et al., 2021), and even directly modulate neuronal activity (Sakry et al., 2014). Given the availability of reporters to label OPCs as well as synaptic and neuronal activity in zebrafish, it will be possible to directly and more deeply interrogate how OPCs affect both neuronal structure and function *in vivo* using zebrafish. As well as determining the currently unrecognised roles of OPCs in larval zebrafish, another exciting area of future study will be determining the functions of OPCs in the adult zebrafish brain (Tsata et al., 2020), both in health and disease.

###### Oligodendrocyte Differentiation

The process of oligodendrocyte differentiation from OPCs is one that can occur by default in the absence of axonal signals (Zeller et al., 1985; Lee et al., 2012; Bechler et al., 2018), upon the activation of gene expression programmes that ultimately converge on building the myelin sheath [reviewed in Czopka and Lyons (2011), Bergles and Richardson (2016), and D’Rozario et al. (2017)]. This default differentiation programme appears to be conserved *in vivo* in zebrafish, as evidence by relatively unaltered oligodendrocyte differentiation observed in mutants with a large reduction in axons fated for myelination (Almeida and Lyons, 2016). As observed in mammals, there appears to be a critical period following differentiation in which oligodendrocytes either commit to myelination, or undergo cell death (Takada and Appel, 2010; Almeida and Lyons, 2016). In general, the molecular regulation of oligodendrocyte differentiation and initiation of myelin gene expression appears well conserved with mammals (Supplementary Table 1). For example, the lysosomal transcription factor Tfeb was recently identified through a forward genetic screen in zebrafish as a negative regulator of myelination, whereby its abrogation leads to premature and excess myelination (Meireles et al., 2018). In parallel, mice lacking TFEB in oligodendrocytes were also found to exhibit precocious and excessive myelination (Sun et al., 2018). In the mouse this was found to be mediated, at least in part, by reduced cell death of newly generated oligodendrocytes. It remains to be determined precisely to what extent the molecular control of oligodendrocyte survival and myelination are distinct or overlap downstream of Tfeb, and how Tfeb and other major transcription factors co-operate to control oligodendrocyte differentiation.

###### Myelination by Oligodendrocytes

Similar to other vertebrates, zebrafish oligodendrocytes make multiple myelin sheaths on many axons upon differentiation (Figure 3), and although some differences in components have been noted the overall molecular composition and ultrastructural nature of myelin in zebrafish and mammals appears broadly similar (Jahn et al., 2009, 2020; Czopka and Lyons, 2011; Preston and Macklin, 2015; Czopka, 2016; Siems et al., 2021) (Figure 3). By live imaging of oligodendrocytes in zebrafish over time using transgenic reporters, Czopka et al. (2013) found that individual cells initiate formation of essentially all of their sheaths within a matter of hours following the initiation of the first sheath, pointing to a restricted period in which oligodendrocytes have to select axons for myelination. Following the selection of axons, the process of myelination continues *via* wrapping of the innermost layer of the myelinating process around and along the axon in a spiralling manner (Snaidero et al., 2014; Nawaz et al., 2015). Following the initial dynamic period of their formation, most sheaths are maintained over time, except for occasional sheath retractions (Czopka et al., 2013; Auer et al., 2018). Whereas initial myelin sheath formation occurs over a matter of hours, imaging studies indicated that subsequent wrapping-based sheath growth occurs over several days, before slowing down and growing in step with the overall growth of the tissue (Auer et al., 2018).

But how are specific axons selected for myelination and why do oligodendrocytes avoid other axons, and indeed other cellular targets for myelination? Almeida et al. (2018) recently found that the relative abundance of oligodendrocytes and axons fated for myelination influences targeting, given that in mutants with fewer axons fated for myelination or in animals with excess oligodendrocytes, oligodendrocytes made myelin around inappropriate targets including neuronal cell bodies. In contrast to myelinating neuronal cell bodies, oligodendrocytes in animals with fewer axons fated for myelination do not myelinate incorrect axons, suggesting more stringent mechanisms preventing their myelination, likely a combination of typically being of smaller axon calibre and expressing inhibitory signals (Klingseisen and Lyons, 2018). Following a forward genetic screen in zebrafish, it was subsequently found that the cell adhesion molecule Neurofascin functions in oligodendrocytes to prevent inappropriate myelination of cell bodies, a mechanism conserved in rodents (Klingseisen et al., 2019). This finding was also made in a parallel study, which implicated the neuronal partner of oligodendrocyte Neurofascin, Contactin1, in mediating correct myelin targeting (Djannatian et al., 2019). Studies in zebrafish have also indicated that certain axons can positively influence myelination (Almeida et al., 2011; Nelson et al., 2020), with many studies now demonstrating that neuronal activity can influence myelination (Nave and Werner, 2014; Baraban et al., 2016; Swire and ffrench-Constant, 2018; Williamson and Lyons, 2018). Studies in zebrafish have contributed to this, with evidence that blocking synaptic vesicle release from all neurons reduces the number of myelin sheaths made by oligodendrocytes and increasing activity increases sheath number (Mensch et al., 2015). Correspondingly, experiments in which the synaptic vesicle release of only some axons was affected indicated that myelination is biased towards higher activity axons (Hines et al., 2015; Koudelka et al., 2016), but interestingly that this only remains true of certain neuronal subtypes (Koudelka et al., 2016), a feature that has more recently also been shown to be true in rodents (Yang et al., 2020).

The mechanisms by which vesicle release regulates myelination was recently investigated by live-cell imaging a transgenic reporter (SypHy) that allows assessment of vesicular release along axons together with a reporter that allows assessment of myelination along the same axons. Somewhat surprisingly, it was found that axonal synaptic vesicle fusion increases upon myelination, and in fact requires myelination. Upon myelination, axonal vesicular fusion becomes enriched adjacent to sites of myelin sheath formation, where it becomes increased upon increasing neuronal activity, which in turn promotes myelination. This led to a feedforward model of activity-regulated myelination, whereby myelination stimulates the localised axonal vesicular release that in turn consolidates myelin sheath growth along axons (Almeida et al., 2021). Although the various molecular mechanisms that regulate the axonal vesicular release remain to be determined, the cell adhesion molecule *N*-cadherin has been proposed to mediate the effects of neuronal activity on myelination in zebrafish (Chen et al., 2017), and may do so by influencing vesicular recycling, as has been shown in mammals (Van Stegen et al., 2017). Recent data has also indicated that axons employ mechanisms that consolidate synapse formation in myelination. This was evidenced by observations that oligodendrocytes in zebrafish can localise the postsynaptic scaffold protein Psd95 to myelin sheaths, and that myelination can be dysregulated by interfering with molecules involved in synapse organisation (Hughes and Appel, 2019; Supplementary Table 1), also seen in mammals (Elazar et al., 2019). In addition to direct signalling between axons and myelinating processes, recent studies have also shown that neuronal activity can influence myelination indirectly, including through regulation of endothelin signalling from the vasculature, which influences myelin sheath production by oligodendrocytes in an Endothelin receptor B dependent manner in both fish and mice (Swire et al., 2019). Future studies will be required to elucidate the potentially multiple activity related axonal signals that influence myelination and the receptors on myelinating processes or on intermediate cells that mediate these effects.

Although the mechanisms of activity-regulated myelination remain to be fully disentangled, two parallel studies in zebrafish indicated that they may influence specific codes of Ca2+ activity in myelin sheaths, that in turn affect myelination. By live imaging individual myelin sheaths, distinct modes of Ca2+ activity were found to prefigure either myelin sheath retractions or myelin sheath elongation (Baraban et al., 2018; Krasnow et al., 2018). Global inhibition of neuronal activity indicated that roughly half of the calcium transients in myelin sheaths were regulated by neuronal firing (Krasnow et al., 2018). A causal role for localised myelin Ca2+ in mediating myelination was evidenced by the finding that localised Ca2+ dependent Calpain protease activity was responsible for driving myelin sheath retractions, Baraban et al. (2018) and that manipulating free Ca2+ levels influenced sheath elongation (Krasnow et al., 2018). How the firing patterns of individual neurons influences distinct Ca2+ activities in myelin sheaths remains to be determined, but is likely to be experimentally tractable in zebrafish by combining optogenetic control of neuronal activity with analyses of corresponding myelin responses. Another mechanism that has been proposed to influence myelination downstream of neuronal activity is that of the local translation of proteins, including myelin structural proteins such as myelin basic protein at the axon-myelin interface (Wake et al., 2011). It has been known for some time that oligodendrocytes transport mRNAs encoding myelin proteins to their distal myelinating processes (Colman et al., 1982), and a forward genetic screen in zebrafish revealed that such mRNA transport in oligodendrocytes is dependent on the kinesin motor protein Kif1b (Lyons et al., 2009). A more recent study has shown that neuronal activity can affect the regulation of mRNAs within myelin sheaths in zebrafish, and that specific 3′UTR elements in *mbp* mRNA regulate its localisation and local translation (Torvund-Jensen et al., 2018). To further investigate the mechanisms that regulate mRNA localisation to myelin sheaths, Yergert et al. (2019) tracked mRNAs as they were transported *in vivo* in the zebrafish. In doing so, distinct 3′UTR elements were identified which direct mRNA to areas of active sheath growth. This study also showed that additional mRNAs have conserved 3′UTR sequences, which appear to drive their localisation to myelin sheaths, including the mRNA encoding the RNA binding fragile X mental retardation protein FMRP, *fmr1* (Yergert et al., 2019). The importance of *fmr1* in directing sheath growth has also been demonstrated in zebrafish, whereby oligodendrocytes lacking *fmr1* have shorter myelin sheaths (Doll et al., 2020), a phenotype that will be important to pursue in the context of understanding circuit function and relevance to developmental disorders.

In addition to mechanisms potentially related to activity-regulated myelination, studies in zebrafish have also provided insight into fundamental cell biology of myelination by oligodendrocyte in the CNS. Live imaging studies in zebrafish placed actin as a major regulator of sheath wrapping, as in mammals (Nawaz et al., 2015; Zuchero et al., 2015), and more recently this has been shown to be influenced by the p21-activated kinase 1 (Pak1) in zebrafish (Brown et al., 2021). In parallel to its role in influencing myelin targeting, oligodendrocyte Neurofascin has been shown to support the growth of myelin sheaths along axons, likely through its interaction with axonal Caspr (Klingseisen et al., 2019). In addition to the regulation of the growth of individual myelin sheaths, the total amount of myelin made by oligodendrocytes is an important factor, and one that has been shown to be regulated by the akt-mtor pathway (Mathews and Appel, 2016a), under the influence of the ubiquitin ligase Fbxw7 (Kearns et al., 2015), a factor that when absent can lead to Schwann cells in the peripheral nervous system myelinating multiple axons like oligodendrocyte through an unknown mechanism independent of its control of Mtor (Harty et al., 2019). Perhaps surprisingly, it remains unclear how the overall production of myelin is regulated over time, an area important for investigation in the future.

##### How Does Myelin Affect Circuit Function?

Myelin sheaths are well known to facilitate rapid action potential propagation along axons, through their lipid-rich, multilamellar composition (Nave and Werner, 2014; Stadelmann et al., 2019), their physiology (Suminaite et al., 2019), and their role in organising key ion channels along axonal subdomains, such as nodes of Ranvier (Sherman and Brophy, 2005; Stassart et al., 2018). In addition, myelin sheaths are thought to represent a conduit for the metabolic support of axons by oligodendrocytes, which has also been proposed to regulate axonal function and integrity (Nave, 2010; Nave and Ehrenreich, 2018) (Figure 3). Furthermore, the fact that myelination can be modified by neuronal activity (Figure 3), and that changes in myelination can affect conduction, has led to the hypothesis that activity regulated myelination may contribute to nervous system plasticity (Fields, 2015; Chang et al., 2016; Almeida and Lyons, 2017; Monje, 2018; Suminaite et al., 2019). The zebrafish offers a unique opportunity to investigate these questions. The first steps towards investigating how CNS myelin in zebrafish affected neural circuit function were however only recently investigated by Madden et al. (2021) in a mutant with disrupted *myrf*, myelin regulatory factor, which affected axonal conduction and zebrafish behaviour. They found that Myrf was required for normal myelination in the CNS (Supplementary Table 1), as in rodents (Bujalka et al., 2013), and that zebrafish *myrf* mutants displayed reduced action potential conduction velocity and have a decreased ability to sustain high frequency action potential firing. To assess the effects of these changes in circuit function on behaviour zebrafish startle responses were studied, which displayed an increased latency to perform startle responses and an aberrant behavioural choice upon sensory stimulation (Madden et al., 2021). Although much remains to be discovered, this study highlights the potential of the zebrafish to integrate electrophysiological protocols with live *in vivo* imaging and behavioural assays, which could also be combined with functional imaging of neuronal and circuit activity to fully interrogate how myelination affects neuronal function and circuit plasticity.

#### Microglia

##### Microglia Formation

**FIGURE 4:**
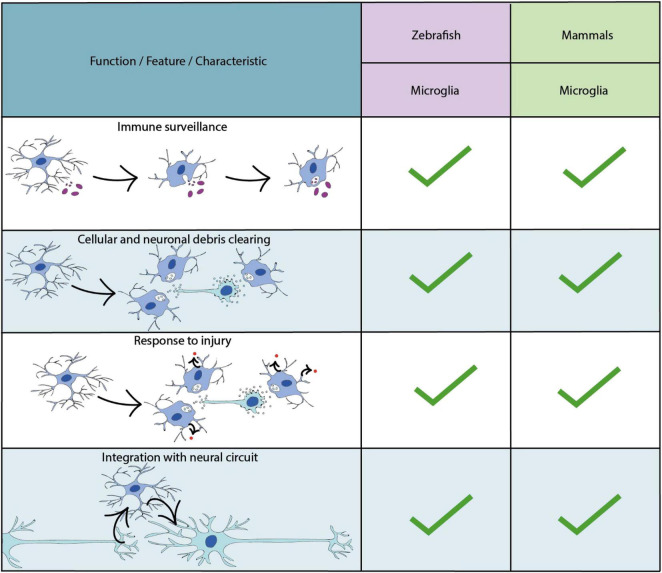
Key characteristics and functional properties of zebrafish and mammalian microglia. The use of “…” refers to areas of microglial biology which require further investigation.

Microglia are the resident immune cells of the central nervous system (Ransohoff and Cardona, 2010) (Figure 4). Recent genetic tracing, live cell imaging and transcriptomic sequencing data has revealed two waves of microglial development within the zebrafish (Xu et al., 2016; Ferrero et al., 2018; Wu et al., 2020) (Figure 1). The first wave originates from a population of primitive macrophages in the yolk sac (Herbomel et al., 2001; Ginhoux et al., 2010; Schulz et al., 2012). These migrate to the hematopoietic tissue [the rostral blood island (RBI)], and then from peripheral tissues to the optic tectum during early embryonic development (Herbomel, 1999). A second wave, which derives from hematopoietic stem cells from the ventral wall of the dorsal aorta replaces the original population later in development (Ferrero et al., 2018; Wu et al., 2020; Silva et al., 2021). A number of conserved transcription factors have been shown to be required for microglia development in zebrafish and mammalian models including Pu.1 and Irf8 (Rhodes et al., 2005; Li et al., 2011; Shiau et al., 2015) (Supplementary Table 1). Additionally, studies in the zebrafish have identified factors essential for the infiltration of yolk sac derived macrophages into the brain. For example, the receptor for macrophage-colony- stimulating factor (Csf1r), has been shown to be essential for microglia migration, as loss of function mutations in both paralogs which encode this receptor induce a complete loss of microglia in the developing zebrafish brain (Herbomel et al., 2001; Wu et al., 2018) (Supplementary Table 1), similar to findings in mammals (Ginhoux et al., 2010). Further dissection of the role of Csf1r using fate-mapping have identified distinct roles for the distinct paralogs of the *csf1r* gene in the zebrafish (Braasch et al., 2006); *csf1ra* and *csf1rb*. Whereas *csf1ra* is important for the initial developmental wave, and later microglia maintenance in the adult zebrafish brain, *csf1rb* has been shown to be essential for colonisation of the CNS by the second microglial wave (Ferrero et al., 2021).

Further studies have also highlighted an essential role for Xpr1, a phosphate exporter, in microglial colonisation of the brain (Meireles et al., 2014), where disruption of both paralogs reduces microglial number in the CNS (Supplementary Table 1). However, unlike the *csf1r* paralogs which independently impair microglial number, *xpr1b* appears to play a much more fundamental role in microglial function than *xpr1a* which was not required for microglial formation. Like the *csf1ra* mutants, *xpr1b* mutants do have a primitive macrophage population but, unlike the *csf1ra* mutation, even after migration into the brain these cells fail to differentiate into microglia, even in later larval developmental stages. Also deriving from a forward genetic screen for genes that regulate microglial formation was the identification of the Nod-like receptor Nlrc3-like gene as being essential for microglial infiltration to the brain. Interesting, Nlrc3l appears to function to downregulate excessive inflammation, whereby mutants exhibited hugely increased inflammation and aggregation of primitive macrophages in the yolk and vasculature preventing their migration to the brain (Shiau et al., 2014) (Supplementary Table 1), bringing up important questions on how peripheral inflammation can regulate CNS microglia, a topic of increasing broad interest.

Upon entry into the CNS, differentiation of microglia is classified based on up-regulation of *apoE*, downregulation of *l-plastin* and a ramified cell morphology (Herbomel et al., 2001), which can be regulated *via* a number of signalling pathways (Yang et al., 2021). Recent work indicates that the process of microglial precursor colonisation of the zebrafish CNS is promoted by programmed neuronal cell death, a normal aspect of early nervous system development in vertebrates (Nijhawan et al., 2000; Dekkers et al., 2013). Interestingly, recent studies in the zebrafish have shown a direct link between the numbers of neurons undergoing apoptosis in different CNS regions and the level of microglial infiltration into them. This was demonstrated by Xu et al. (2016) who showed that inhibition of neuronal cell death reduces the number of microglial precursors which enter the optic tectum in the developing zebrafish. To investigate the signals which may mediate this process, Casano et al. (2016) utilised a photoconvertible protein expressed in microglia to track the progress of microglia in a system with inducible neuronal cell death. Enhanced infiltration of microglia into specific regions with significant neuronal apoptosis was shown to be mediated by nucleotide-mediated chemotaxis, which has also been reported in murine studies (Haynes et al., 2006), where nucleotide release is associated with cell or tissue damage. Taken together these data demonstrate that neuronal apoptosis mediates microglial CNS infiltration into specific regions, which is used during zebrafish development to colonise the CNS and influence microglia number.

##### Functional Roles of Microglia

The process by which microglia engulf dying neurons has been investigated in the zebrafish by time-lapse imaging (Peri and Nüsslein-Volhard, 2008). Neuronal engulfment is reliant on combined interactions between Tim-4 and Bai1, two phosphatidylserine receptors on the microglial surface (Supplementary Table 1). Specifically, Bai1 is involved in phagosome formation and transport whilst Tim-4 is necessary for phagosome stabilisation *via* actin polymerisation (Mazaheri et al., 2014). Once phagocytosed, the Rag-Regulator complex is essential in the formation of lysosomes and efficient digestion of neuronal debris (Shen et al., 2016). Further investigations have added to the list of phagocytic signals that could prompt engulfment of dying neurons by microglial cells in the zebrafish including lysophosphatidylcholine, a phospholipid known to be released from apoptotic neurons (Xu et al., 2016) which is also a potent chemoattractant for microglia in rodents.

But how can microglia coordinate their migration towards phagocytic targets, and how are microglia recruited long distances throughout the CNS? One candidate signalling pathway, which has been shown to facilitate long range migration of microglia to damaged neurons is calcium signalling. Utilising a targeted laser neuronal ablation approach to damage neurons in the brains of larval fish expressing calcium reporters in microglia Sieger et al. (2012) observed rapid calcium waves which determined the direction and magnitude of the microglial response, as killing fewer neurons resulted in shorter range Ca2+ waves and migration of local microglia only. Furthermore, preventing the formation of such Ca2+ waves or chelating extracellular Ca2+ reduced microglia migration to sites of damage, suggesting that Ca2+ signalling plays an essential role in neuronal-microglial interactions. But how could neuronal signalling influence the generation of Ca2+ waves within microglia? Interestingly, the generation of Ca2+ waves was found to be dependent on glutamate and NMDA-receptor signalling, which may facilitate communication between neuronal and microglial cells in a number of physiological as well as disease/injury situations, given that microglial contacts with neurons have been shown to be affected by neuronal activity (Hughes and Appel, 2020). Microglia have also been implicated in synapse formation and function including synaptic pruning and removal of inappropriate synaptic contacts in mammals, which is a normal aspect of vertebrate development. In the zebrafish the contribution of glia to synapse formation, strengthening and pruning is far less extensively studied than in mammals, and remains an exciting area for future research. That being said, there is evidence to suggest that microglia in the zebrafish may play important roles in synaptogenesis. For example, a recent study from Silva et al. (2021) describe a novel subpopulation of synapse-associated microglia present in the midbrain and hindbrain. Synapse-associated microglia express the complement gene C1qc, a known protein which also regulates synaptic engulfment by microglia in rodents (Stevens et al., 2007; Hong et al., 2016), which could suggest that it may mediate similar functions in the zebrafish.

Microglia can also regulate homeostatic neuronal activity in zebrafish. This was explored in a study by Li et al. (2012) who integrated *in vivo* imaging of microglial cells, whole-cell electrophysiology recording, and glutamate uncaging. By monitoring microglial morphology, process dynamics and neuronal activity they showed that local increases in neuronal activity attracted microglial processes, which contacted the somas of highly active neuronal cells. Once microglia-neuron contacts were established after about 5 min this contact downregulated spontaneous activity in the neuron, which remained at a low level for at least 7 min even after the interaction. Further mechanistic investigation showed that this process was dependent on pannexin-1 hemichannels on the neuronal soma, triggering the release of signals which attract microglia *via* activation of the small Rho GTPase Rac in resting microglia (Li et al., 2012). Intriguingly, a recent study utilising a mouse model has provided further evidence that microglia may mediate negative feedback mechanisms to protect the brain from excessive activity, highlighting a conserved feature of microglia biology across species (Badimon et al., 2021). Together these results raise fascinating questions about the roles of microglia in regulating neural excitation in the healthy CNS, the potential roles of microglia at synapses, and the effects these may have on neuronal function if such interactions become perturbed in disease.

Interestingly, neuronal regulation of microglial function has recently been demonstrated to include microglial phagocytosis of non-neuronal cells. For example, microglia have recently been proposed to be capable of phagocytosing myelin sheaths during development. Similar to synapses and neurons, more myelin sheaths are initially produced in development than are eventually maintained over time. Whilst much of this was demonstrated to be driven by oligodendrocytes retracting myelin sheaths, as visualised *in vivo* in the zebrafish, Hughes and colleagues have recently described a contribution of microglia to developmental myelin sheath elimination. By studying the interactions of microglia with myelinated axons, they observed close association of microglia with both neuronal somas and myelinated regions of the axon (Hughes and Appel, 2020). Very interestingly, in animals with fewer microglia, individual oligodendrocytes had a greater number of myelin sheaths, without any significant effect on oligodendrocyte number, directly implicating microglia in the control of myelination by single oligodendrocytes (Hughes and Appel, 2020). This data raises a number of questions regarding the potential impacts of microglia in life long myelin plasticity and refinement of neural circuit structure and function, which await investigation. Microglia remain in the CNS throughout life with the ability to quickly transition into distinct functional states. Given that recent gene expression profiling studies have shown larval zebrafish microglia to have a conserved microglia signature *in vivo* compared to other model organisms, this places the zebrafish in a central position for future study of these cells (Mazzolini et al., 2019), in the healthy nervous system, but also in various disease modelling contexts, which are becoming established in zebrafish.

## Discussion

The current landscape of zebrafish glial research is an exciting one given the rapid advances in genetic manipulation strategies, imaging techniques, probes for labelling, fluorescent reporters, ablation techniques, optogenetics, drug screening and the ability to integrate many of these whilst also studying behavioural responses. Additionally, advances in cell type specific investigation of gene function, and platforms to simultaneously investigate glial and neuronal function within the context of behavioural outputs will, no doubt, place the zebrafish in a central position for future investigation of glial cells in health and disease.

Recent research has highlighted the diversity of functions played by glia in the zebrafish, and how their similarities with other model organisms can be used to identify conserved biological pathways. Moreover, differences between the highly regenerative zebrafish and other organisms provide a useful comparison where novel pathways with potential implications in repair can be investigated. Given the range of essential roles that glia perform in development, circuit function and lifelong maintenance of the healthy nervous system, it follows that disruption to glia, or changes to the functional roles which they play can characterise disease. The zebrafish, as a model organism, is emerging as a popular choice to study glial cells in disease and regeneration. For example, the roles of glial cells in epileptic seizures, brain tumours, degenerative disease, remyelination, peripheral nerve regeneration, retinal regeneration, and spinal cord repair are now being studied in zebrafish models of disease (Mokalled et al., 2016; Sifuentes et al., 2016; Karttunen et al., 2017; Chia et al., 2018, 2019; Frøyset et al., 2018; Saleem and Kannan, 2018; Diaz Verdugo et al., 2019; Neely et al., 2020). Given the applications of zebrafish to investigate glial cells throughout life, in disease and for screening applications we believe that this makes for exciting future research into glial cell function to complement that in other models.

## Author Contributions

Both authors listed have made a substantial, direct, and intellectual contribution to the work, and approved it for publication.

## Conflict of Interest

The authors declare that the research was conducted in the absence of any commercial or financial relationships that could be construed as a potential conflict of interest.

## Publisher’s Note

All claims expressed in this article are solely those of the authors and do not necessarily represent those of their affiliated organizations, or those of the publisher, the editors and the reviewers. Any product that may be evaluated in this article, or claim that may be made by its manufacturer, is not guaranteed or endorsed by the publisher.

## References

[B1] Ablain J. Durand E. M. Yang S. Zhou Y. Zon L. I. (2015). A CRISPR/Cas9 vector system for tissue-specific gene disruption in zebrafish. *Dev. Cell* 32 756–764. 10.1016/J.DEVCEL.2015.01.032 25752963 PMC4379706

[B2] Ackerman S. D. Monk K. R. (2016). The scales and tales of myelination: using zebrafish and mouse to study myelinating glia. *Brain Res.* 1641 79–91. 10.1016/j.brainres.2015.10.011 26498880 PMC4838556

[B3] Ackerman S. D. Garcia C. Piao X. Gutmann D. H. Monk K. R. (2015). The adhesion GPCR Gpr56 regulates oligodendrocyte development via interactions with Gα12/13 and RhoA. *Nat. Commun.* 6 1–14. 10.1038/ncomms7122 25607772 PMC4302765

[B4] Ahrens M. B. Orger M. B. Robson D. N. Li J. M. Keller P. J. (2013). Whole-brain functional imaging at cellular resolution using light-sheet microscopy. *Nat. Methods* 10 413–420. 10.1038/nmeth.2434 23524393

[B5] Akay L. A. Effenberger A. H. Tsai L. H. (2021). Cell of all trades: oligodendrocyte precursor cells in synaptic, vascular, and immune function. *Genes Dev.* 35 180–198. 10.1101/GAD.344218.120 33526585 PMC7849363

[B6] Ali M. F. Latimer A. J. Wang Y. Hogenmiller L. Fontenas L. Isabella A. J. (2021). Met is required for oligodendrocyte progenitor cell migration in *Danio rerio*. *bioRxiv* [Preprint] 10.1101/2021.05.21.445204 PMC8473979 34568921

[B7] Allen N. J. Eroglu C. (2017). Cell biology of astrocyte-synapse interactions. *Neuron* 96 697–708. 10.1016/J.NEURON.2017.09.056 29096081 PMC5687890

[B8] Allen N. J. Lyons D. A. (2018). System formation and function. *Science* 185 181–185. 10.1126/science.aat0473 PMC6292669 30309945

[B9] Almeida R. G. Lyons D. A. (2017). On myelinated axon plasticity and neuronal circuit formation and function. *J. Neurosci.* 37 10023–10034. 10.1523/jneurosci.3185-16.2017 29046438 PMC6596541

[B10] Almeida R. G. Czopka T. ffrench-Constant C. Lyons D. A. (2011). Individual axons regulate the myelinating potential of single oligodendrocytes in vivo. *Development* 138 4443–4450. 10.1242/dev.071001 21880787 PMC3177314

[B11] Almeida R. G. Pan S. Cole K. L. H. Williamson J. M. Early J. J. Czopka T. (2018). Myelination of neuronal cell bodies when myelin supply exceeds axonal demand. *Curr. Biol.* 28 1296–1305.e5. 10.1016/j.cub.2018.02.068 29628374 PMC5912901

[B12] Almeida R. G. Williamson J. M. Madden M. E. Talbot W. S. Bianco I. H. Lyons D. A. (2021). Myelination induces axonal hotspots of synaptic vesicle fusion that promote sheath growth. *Curr. Biol.* 31 1–12. 10.1016/j.cub.2021.06.036 34270947 PMC8445327

[B13] Almeida R. Lyons D. (2016). Oligodendrocyte development in the absence of their target axons in Vivo. *PLoS One* 11:1–24. 10.1371/journal.pone.0164432 27716830 PMC5055324

[B14] Andalman A. S. Burns V. M. Lovett-Barron M. Broxton M. Poole B. Yang S. J. (2019). Neuronal dynamics regulating brain and behavioral state transitions. *Cell* 177 970–985. 10.1016/j.cell.2019.02.037 31031000 PMC6726130

[B15] Antinucci P. Dumitrescu A. S. Deleuze C. Morley H. J. Leung K. Hagley T. (2020). A calibrated optogenetic toolbox of stable zebrafish opsin lines. *elife* 9:e54937. 10.7554/eLife.54937 32216873 PMC7170653

[B16] Appel B. Givan L. A. Eisen J. S. (2001). Delta-Notch signaling and lateral inhibition in zebrafish spinal cord development. *BMC Dev. Biol.* 1:1–11. 10.1186/1471-213X-1-13 11495630 PMC37243

[B17] Arrenberg A. B. Del Bene F. Baier H. (2009). Optical control of zebrafish behavior with halorhodopsin. *Proc. Natl. Acad. Sci. U.S.A.* 106 17968–17973. 10.1073/PNAS.0906252106 19805086 PMC2764931

[B18] Auer F. Vagionitis S. Czopka T. (2018). Evidence for myelin sheath remodeling in the CNS revealed by in vivo imaging. *Curr. Biol.* 28 549–559. 10.1016/j.cub.2018.01.017 29429620

[B19] Badimon A. Strasburger H. J. Ayata P. Chen X. Ikegami A. Hwang P. (2021). Negative feedback control of neuronal activity by microglia. *Nature* 586 417–423. 10.1038/s41586-020-2777-8.Negative PMC7577179 32999463

[B20] Baraban M. Koudelka S. Lyons D. A. (2018). Ca 2+ activity signatures of myelin sheath formation and growth in vivo. *Nat. Neurosci.* 21 19–25. 10.1038/s41593-017-0040-x 29230058 PMC5742537

[B21] Baraban M. Mensch S. Lyons D. A. (2016). Adaptive myelination from fish to man. *Brain Res.* 1641 149–161. 10.1016/j.brainres.2015.10.026 26498877 PMC4907128

[B22] Barres B. A. (2008). The mystery and magic of glia: a perspective on their roles in health and disease. *Neuron* 60 430–440. 10.1016/j.neuron.2008.10.013 18995817

[B23] Bechler M. E. Swire M. ffrench-Constant C. (2018). Intrinsic and adaptive myelination—a sequential mechanism for smart wiring in the brain. *Dev. Neurobiol.* 78 68–79. 10.1002/dneu.22518 28834358 PMC5813148

[B24] Bellini M. J. Suk K. Steardo L. Carvalho F. Gomes A. Matias I. (2019). Astrocyte heterogeneity: impact to brain aging and disease. *Front. Aging Neurosci.* 11:59. 10.3389/fnagi.2019.00059 30941031 PMC6433753

[B25] Bergles D. E. Richardson W. D. (2016). Oligodendrocyte development and plasticity. *Cold Spring Harb. Perspect. Biol.* 8 1–27. 10.1101/cshperspect.a020453 26492571 PMC4743079

[B26] Bernardos R. L. Raymond P. A. (2006). GFAP transgenic zebrafish. *Gene Expr. Patterns* 6 1007–1013. 10.1016/j.modgep.2006.04.006 16765104

[B27] Bianco I. H. Engert F. Bianco I. H. Engert F. (2015). Visuomotor transformations underlying hunting behavior in zebrafish article visuomotor transformations underlying hunting behavior in zebrafish. *Curr. Biol.* 25 831–846. 10.1016/j.cub.2015.01.042 25754638 PMC4386024

[B28] Bianco I. H. Kampff A. R. Engert F. Stephan C. F. (2011). Prey capture behavior evoked by simple visual stimuli in larval zebrafish. *Front. Syst. Neurosci.* 5:101. 10.3389/fnsys.2011.00101 22203793 PMC3240898

[B29] Bin J. M. Lyons D. A. (2016). Imaging myelination in vivo using transparent animal models. *Brain Plast.* 2 3–29. 10.3233/bpl-160029 29765846 PMC5928531

[B30] Bowen M. E. Henke K. Siegfried K. R. Warman M. L. Harris M. P. (2012). Efficient mapping and cloning of mutations in zebrafish by low-coverage whole-genome sequencing. *Genetics* 190 1017–1024. 10.1534/genetics.111.136069 22174069 PMC3296239

[B31] Braasch I. Salzburger W. Meyer A. (2006). Asymmetric evolution in two fish-specifically duplicated receptor tyrosine kinase paralogons involved in teleost coloration. *Mol. Biol. Evol.* 23 1192–1202. 10.1093/molbev/msk003 16547150

[B32] Bremer J. Skinner J. Granato M. (2017). A small molecule screen identifies in vivo modulators of peripheral nerve regeneration in zebrafish. *PLoS One* 12:1–17. 10.1371/journal.pone.0178854 28575069 PMC5456414

[B33] Brenner S. (2003). The genetics of caenorhabditis elegans. *Genetics* 4 683–687. 10.1002/cbic.200300625 4366476 PMC1213120

[B34] Brown T. L. Hashimoto H. Finseth L. T. Wood T. L. MacKlin W. B. (2021). Pak1 positively regulates oligodendrocyte morphology and myelination. *J. Neurosci.* 41 1864–1877. 10.1523/JNEUROSCI.0229-20.2021 33478987 PMC7939082

[B35] Buchanan J. Elabbady L. Collman F. Jorstad N. L. Bakken T. E. Ott C. (2021). Oligodendrocyte precursor cells prune axons in the mouse neocortex. *bioRxiv* [Preprint] 10.1101/2021.05.29.446047 PMC9889886 36417438

[B36] Buckley C. E. Marguerie A. Roach A. G. Goldsmith P. Fleming A. Alderton W. K. (2010). Drug reprofiling using zebrafish identifies novel compounds with potential pro-myelination effects. *Neuropharmacology* 59 149–159. 10.1016/j.neuropharm.2010.04.014 20450924

[B37] Buckley C. E. Moore R. E. Reade A. Goldberg A. R. Weiner O. D. Clarke J. D. W. (2016). Reversible optogenetic control of subcellular protein localization in a live vertebrate embryo. *Dev. Cell* 36 117–126. 10.1016/j.devcel.2015.12.011 26766447 PMC4712025

[B38] Bujalka H. Koenning M. Jackson S. Perreau V. M. Pope B. Hay C. M. (2013). MYRF Is a membrane-associated transcription factor that autoproteolytically cleaves to directly activate myelin genes. *PLoS Biol.* 11: e1001625. 10.1371/journal.pbio.1001625 23966833 PMC3742440

[B39] Bushong E. A. Martone M. E. Jones Y. Z. Ellisman M. H. (2002). Protoplasmic astrocytes in CA1 stratum radiatum occupy separate anatomical domains. *J. Neurosci.* 22 183–192. 10.1523/jneurosci.22-01-00183.2002 11756501 PMC6757596

[B40] Casano A. M. Albert M. Peri F. (2016). Developmental apoptosis mediates entry and positioning of microglia in the zebrafish brain. *Cell Rep.* 16 897–906. 10.1016/j.celrep.2016.06.033 27425604

[B41] Cayre M. Falque M. Mercier O. Magalon K. Durbec P. (2021). Myelin repair: from animal models to humans. *Front. Cell. Neurosci.* 15: 604865. 10.3389/FNCEL.2021.604865 33935649 PMC8079744

[B42] Chang K.-J. Redmond S. A. Chan J. R. (2016). Remodeling myelination: implications for mechanisms of neural plasticity. *Nat. Neurosci.* 192 190–197. 10.1038/nn.4200 26814588 PMC4792270

[B43] Charlton-Perkins M. Almeida A. D. MacDonald R. B. Harris W. A. Ryan MacDonald C. B. (2019). Genetic control of cellular morphogenesis in Müller glia. *Glia* 67 1401–1411. 10.1002/glia.23615 30924555 PMC6563441

[B44] Chavali M. Ulloa-Navas M. J. Pérez-Borredá P. Garcia-Verdugo J. M. McQuillen P. S. Huang E. J. (2020). Wnt-dependent oligodendroglial-endothelial interactions regulate white matter vascularization and attenuate injury. *Neuron* 108 1130–1145.e5. 10.1016/j.neuron.2020.09.033 33086038 PMC7769920

[B45] Chen J. Poskanzer K. E. Freeman M. R. Monk K. R. (2020). Live-imaging of astrocyte morphogenesis and function in zebrafish neural circuits. *Nat. Neurosci.* 23 1297–1306. 10.1038/s41593-020-0703-x 32895565 PMC7530038

[B46] Chen M. Xu Y. Huang R. Huang Y. Ge S. Hu B. (2017). N-Cadherin is involved in neuronal activity-dependent regulation of myelinating capacity of zebrafish individual oligodendrocytes in vivo. *Mol. Neurobiol.* 54 6917–6930. 10.1007/s12035-016-0233-4 27771903

[B47] Chia K. Keatinge M. Mazzolini J. Sieger D. (2019). Brain tumours repurpose endogenous neuron to microglia signalling mechanisms to promote their own proliferation. *elife* 8:e46912. 10.7554/eLife.46912 PMC6685703 31313988

[B48] Chia K. Mazzolini J. Mione M. Sieger D. (2018). Tumor initiating cells induce Cxcr4- mediated infiltration of pro-tumoral macrophages into the brain. *elife* 7 1–28. 10.7554/eLife.31918 PMC5821457 29465400

[B49] Cho W. Barcelon E. Lee S. J. (2016). Optogenetic glia manipulation?: possibilities and future prospects. *Exp. Neurobiol.* 25 197–204. 27790054 10.5607/en.2016.25.5.197 PMC5081466

[B50] Chung W. Allen N. J. Eroglu C. (2015). Astrocyte control synapse formation, function and elimination. *Cold Spring Harb. Lab. Press* 7 a020370. 10.1101/cshperspect.a020370 PMC4527946 25663667

[B51] Clarke L. E. Barres B. A. (2013). Emerging roles of astrocytes in neural circuit development. *Nat. Rev. Neurosci.* 14 311–321. 10.1038/nrn3484 23595014 PMC4431630

[B52] Clayton B. L. L. Tesar P. J. (2021). Oligodendrocyte progenitor cell fate and function in development and disease. *Curr. Opin. Cell Biol.* 73 35–40. 10.1016/j.ceb.2021.05.003 34153742 PMC8678156

[B53] Clemente D. Ortega M. C. Melero-Jerez C. De Castro F. (2013). The effect of glia-glia interactions on oligodendrocyte precursor cell biology during development and in demyelinating diseases. *Front. Cell. Neurosci.* 7:268. 10.3389/FNCEL.2013.00268 24391545 PMC3868919

[B54] Colman D. R. Kreibich G. Frey A. B. Sabatini D. D. (1982). Synthesis and incorporation of myelin polypeptides into CNS myelin. *J. Cell Biol.* 95 598–608. 10.1083/JCB.95.2.598 6183276 PMC2112951

[B55] Cong L. Wang Z. Chai Y. Hang W. Shang C. Yang W. (2017). Rapid whole brain imaging of neural activity in freely behaving larval zebrafish (*Danio rerio*). *elife* 6 e28158. 10.7554/eLife.28158 28930070 PMC5644961

[B56] Cornet C. Di Donato V. Terriente J. (2018). Combining zebrafish and CRISPR/Cas9: toward a more efficient drug discovery pipeline. *Front. Pharmacol.* 9:703. 10.3389/FPHAR.2018.00703 30018554 PMC6037853

[B57] Cuoghi B. Mola L. (2009). Macroglial cells of the teleost central nervous system: a survey of the main types. *Cell Tissue Res.* 338 319–332. 10.1007/s00441-009-0870-2 19865831

[B58] Czopka T. (2016). Insights into mechanisms of central nervous system myelination using zebrafish. *Glia* 64 333–349. 10.1002/glia.22897 26250418

[B59] Czopka T. Lyons D. A. (2011). *Dissecting Mechanisms of Myelinated Axon Formation Using Zebrafish Third Edit.* Amsterdam: Elsevier Inc, 10.1016/B978-0-12-381320-6.00002-3 21951525

[B60] Czopka T. ffrench-Constant C. Lyons D. A. (2013). Individual oligodendrocytes have only a few hours in which to generate new myelin sheaths invivo. *Dev. Cell* 25 599–609. 10.1016/j.devcel.2013.05.013 23806617 PMC4013507

[B61] D’Rozario M. Monk K. R. Petersen S. C. (2017). *Analysis of Myelinated Axon Formation in Zebrafish.* Amsterdam: Elsevier Ltd, 10.1016/bs.mcb.2016.08.001 PMC5661983 28129853

[B62] Dallérac G. Zapata J. Rouach N. (2018). Versatile control of synaptic circuits by astrocytes: where, when and how? *Nat. Rev. Neurosci.* 1912 729–743. 10.1038/s41583-018-0080-6 30401802

[B63] Dekkers M. P. J. Nikoletopoulou V. Barde Y. A. (2013). Death of developing neurons: new insights and implications for connectivity. *J. Cell Biol.* 203 385–393. 10.1083/jcb.201306136 24217616 PMC3824005

[B64] Demy D. Lou, Carrere M. Noche R. Tauzin M. Le Bris M. (2021). The cationic amino acid exporter Slc7a7 is induced and vital in zebrafish tissue macrophages with sustained efferocytic activity. *J. Cell Sci.* 133 1–11. 10.1242/jcs.249037 32973110

[B65] Diaz Verdugo C. Myren-Svelstad S. Aydin E. Van Hoeymissen E. Deneubourg C. Vanderhaeghe S. (2019). Glia-neuron interactions underlie state transitions to generalized seizures. *Nat. Commun.* 10: 3830. 10.1038/s41467-019-11739-z 31444362 PMC6707163

[B66] Ding F. O’Donnell J. Thrane A. S. Zeppenfeld D. Kang H. Xie L. (2013). α1-Adrenergic receptors mediate coordinated Ca2+ signaling of cortical astrocytes in awake, behaving mice. *Cell Calcium* 54 387–394. 10.1016/j.ceca.2013.09.001 24138901 PMC3858490

[B67] Djannatian M. Timmler S. Arends M. Luckner M. Weil M.-T. Alexopoulos I. (2019). Two adhesive systems cooperatively regulate axon ensheathment and myelin growth in the CNS. *Nat. Commun.* 10:4794. 10.1038/s41467-019-12789-z 31641127 PMC6805957

[B68] Doll C. A. Scott K. Appel B. (2021). Fmrp regulates oligodendrocyte lineage cell specification and differentiation. *bioRxiv* [Preprint] 10.1101/2021.03.16.435661 PMC8373694 34110049

[B69] Doll C. A. Yergert K. M. Appel B. H. (2020). The RNA binding protein fragile X mental retardation protein promotes myelin sheath growth. *Glia* 68 495–508. 10.1002/glia.23731 31626382 PMC8279157

[B70] Driever W. Stemple D. (1994). Zebrafish?: genetic tools for studying vertebrate development. *Trends Genet.* 10 152–159. 8036717 10.1016/0168-9525(94)90091-4

[B71] Driever W. Solnica-Krezel L. Schier A. F. Neuhauss S. C. F. Malicki J. Stemple D. L. (1996). A genetic screen for mutations affecting embryogenesis in zebrafish. *Development* 123 37–46. 10.1242/dev.123.1.37 9007227

[B72] Early J. J. Cole K. L. Williamson J. M. Swire M. Kamadurai H. Muskavitch M. (2018). An automated high-resolution in vivo screen in zebrafish to identify chemical regulators of myelination. *elife* 7:e35136. 10.7554/elife.35136 29979149 PMC6056238

[B73] Eastlake K. Lamb W. D. B. Luis J. Khaw P. T. Jayaram H. Limb G. A. (2021). Prospects for the application of *Müller glia* and their derivatives in retinal regenerative therapies. *Prog. Retin. Eye Res.* 10:100970. 10.1016/j.preteyeres.2021.100970 33930561

[B74] Elazar N. Vainshtein A. Golan N. Vijayaragavan B. Schaeren-Wiemers N. Eshed-Eisenbach Y. (2019). Axoglial adhesion by CADM4 regulates CNS myelination. *Neuron* 101 224–231.e5. 10.1016/j.neuron.2018.11.032 30551998 PMC6371057

[B75] Engert F. (2012). Fish in the matrix: motor learning in a virtual world. *Front. Neural Circuits* 6:125. 10.3389/fncir.2012.00125 23355810 PMC3555039

[B76] Erskine L. Herrera E. (2007). The retinal ganglion cell axon’s journey: insights into molecular mechanisms of axon guidance. *Dev. Biol.* 308 1–14. 10.1016/j.ydbio.2007.05.013 17560562

[B77] Fairchild C. L. Hino K. Han J. S. Miltner A. M. Peinado Allina G. Brown C. E. (2018). RBX2 maintains final retinal cell position in a DAB1-dependent and -independent fashion. *Development* 145:dev155283. 10.1242/DEV.155283 29361558 PMC5817999

[B78] Falk S. Götz M. (2017). Glial control of neurogenesis. *Curr. Opin. Neurobiol.* 47 188–195. 10.1016/j.conb.2017.10.025 29145015

[B79] Farhy-Tselnicker I. Allen N. J. (2018). Astrocytes, neurons, synapses: a tripartite view on cortical circuit development. *Neural Dev.* 13 1–12. 10.1186/s13064-018-0104-y 29712572 PMC5928581

[B80] Farnsworth D. R. Saunders L. M. Miller A. C. (2020). A single-cell transcriptome atlas for zebrafish development. *Dev. Biol.* 459 100–108. 10.1016/j.ydbio.2019.11.008 31782996 PMC7080588

[B81] Fausett B. V. Gumerson J. D. Goldman D. (2008). The Proneural basic helix-loop-helix gene ASCL1A is required for retina regeneration. *J. Neurosci.* 28 1109–1117. 10.1523/JNEUROSCI.4853-07.2008 18234889 PMC2800945

[B82] Ferrero G. Mahony C. B. Dupuis E. Yvernogeau L. Di Ruggiero E. Miserocchi M. (2018). Embryonic microglia derive from primitive macrophages and are replaced by cmyb-dependent definitive microglia in zebrafish. *Cell Rep.* 24 130–141. 10.1016/j.celrep.2018.05.066 29972775

[B83] Ferrero G. Miserocchi M. Di Ruggiero E. Wittamer V. (2021). A csf1rb mutation uncouples two waves of microglia development in zebrafish. *Devlopment* 148 1–12. 10.1242/dev.194241 33298459

[B84] Fields R. D. (2015). A new mechanism of nervous system plasticity: activity-dependent myelination. *Nat. Rev. Neurosci.* 16 756–767. 10.1038/NRN4023 26585800 PMC6310485

[B85] Fontenas L. Kucenas S. (2018). Motor exit point (MEP) glia: novel myelinating glia that bridge CNS and PNS myelin. *Front. Cell. Neurosci.* 12:1–8. 10.3389/fncel.2018.00333 30356886 PMC6190867

[B86] Franklin R. J. M. Frisén J. Lyons D. A. (2020). Revisiting remyelination: towards a consensus on the regeneration of CNS myelin. *Semin. Cell Dev. Biol.* 116 3–9. 10.1016/j.semcdb.2020.09.009 33082115

[B87] Freeman M. R. (2010). Specification and morphogenesis of astrocytes. *Science* 330 774–778. 10.1126/science.1190928 21051628 PMC5201129

[B88] Freeman M. R. Doherty J. (2006). Glial cell biology in *Drosophila* and vertebrates. *Trends Neurosci.* 29 82–90. 10.1016/j.tins.2005.12.002 16377000

[B89] Frøyset A. K. Edson A. J. Gharbi N. Khan E. A. Dondorp D. Bai Q. (2018). Astroglial DJ-1 over-expression up-regulates proteins involved in redox regulation and is neuroprotective in vivo. *Redox Biol.* 16 237–247. 10.1016/j.redox.2018.02.010 29525604 PMC5854894

[B90] Gaj T. Gersbach C. A. Barbas C. F. (2013). ZFN, TALEN, and CRISPR/Cas-based methods for genome engineering. *Trends Biotechnol.* 31 397–405. 10.1016/j.tibtech.2013.04.004 23664777 PMC3694601

[B91] Gan L. Seki A. Shen K. Iyer H. Han K. Hayer A. (2019). The lysosomal GPCR-like protein GPR137B regulates Rag and mTORC1 localization and activity. *Nat. Cell Biol.* 21 614–626. 10.1038/s41556-019-0321-6 31036939 PMC6649673

[B92] García-García D. Locker M. Perron M. (2020). Update on *Müller glia* regenerative potential for retinal repair. *Curr. Opin. Genet. Dev.* 64 52–59. 10.1016/j.gde.2020.05.025 32619816

[B93] Geraghty A. C. Gibson E. M. Ghanem R. A. Greene J. J. Ocampo A. Goldstein A. K. (2019). Loss of adaptive myelination contributes to methotrexate chemotherapy-related cognitive impairment HHS Public Access. *Neuron* 103 250–265. 10.1016/j.neuron.2019.04.032 31122677 PMC6697075

[B94] Ginhoux F. Greter M. Leboeuf M. Nandi S. See P. Gokhan S. (2010). Fate mapping analysis reveals that adult microglia derive from primitive macrophages. *Science* 701 841–845. 10.1126/science.1194637 PMC3719181 20966214

[B95] Goldberg J. L. Vargas M. E. Wang J. T. Mandemakers W. Oster S. F. Sretavan D. W. (2004). An oligodendrocyte lineage-specific semaphorin, sema5A, inhibits axon growth by retinal ganglion cells. *J. Neurosci.* 24 4989–4999. 10.1523/JNEUROSCI.4390-03.2004 15163691 PMC6729380

[B96] Goldman D. (2014). *Müller glia* cell reprogramming and retina regeneration. *Nat. Rev. Neurosci.* 15 431–442. 10.1038/nrn3723.M 24894585 PMC4249724

[B97] Gorsuch R. A. Lahne M. Yarka C. E. Petravick M. E. Li J. Hyde D. R. (2017). Sox2 regulates *Müller glia* reprogramming and proliferation in the regenerating zebra fish retina via Lin28 and Ascl1a. *Exp. Eye Res.* 161 174–192. 10.1016/j.exer.2017.05.012 28577895 PMC5554723

[B98] Götz M. Sirko S. Beckers J. Irmler M. (2015). Reactive astrocytes as neural stem or progenitor cells: in vivo lineage, in vitro potential, and genome-wide expression analysis. *Glia* 63 1452–1468. 10.1002/GLIA.22850 25965557 PMC5029574

[B99] Grupp L. Wolburg H. Mack A. F. (2010). Astroglial structures in the zebrafish brain. *J. Comp. Neurol.* 518 4277–4287. 10.1002/cne.22481 20853506

[B100] Haffter P. Granato M. Brand M. Mullins M. C. Hammerschmidt M. Kane D. A. (1996). The identification of genes with unique and essential functions in the development of the zebrafish *Danio rerio*. *Development* 123 1–36. 10.1242/dev.123.1.1 9007226

[B101] Hammond B. P. Manek R. Kerr B. J. Macauley M. S. Plemel J. R. (2021). Regulation of microglia population dynamics throughout development, health, and disease. *Glia* 69, 2771–2797. 10.1002/glia.24047 34115410

[B102] Hamon A. Roger J. E. Yang X. J. Perron M. (2016). Müller glial cell-dependent regeneration of the neural retina: an overview across vertebrate model systems. *Dev. Dyn.* 245 727–738. 10.1002/dvdy.24375 26661417 PMC4900950

[B103] Hans S. Zöller D. Hammer J. Stucke J. Spieß S. Kesavan G. (2021). Cre-Controlled CRISPR mutagenesis provides fast and easy conditional gene inactivation in zebrafish. *Nat. Commun.* 12 1–12. 10.1038/s41467-021-21427-6 33602923 PMC7893016

[B104] Harboe M. Torvund-Jensen J. Kjaer-Sorensen K. Laursen L. S. (2018). Ephrin-A1-EphA4 signaling negatively regulates myelination in the central nervous system. *Glia* 66 934–950. 10.1002/glia.23293 29350423

[B400] Harlow D. E. Saul K. E. Komuro H. Macklin W. B. (2015). Myelin proteolipid protein complexes with αv integrin and AMPA receptors *in vivo* and regulates AMPA-dependent oligodendrocyte progenitor cell migration through the modulation of cell-surface gluR2 expression. *J. Neurosci.* 35, 12018–12032. 10.1523/JNEUROSCI.5151-14.2015 26311781 PMC4549408

[B105] Harrington E. Bergles D. E. Calabresi P. A. (2020). Immune cell modulation of oligodendrocyte lineage cells. *Neurosci. Lett.* 715 1–9. 10.1016/J.NEULET.2019.134601 31693930 PMC6981227

[B106] Harris J. M. Wang A. Y.-D. Boulanger-Weill J. Santoriello C. Foianini S. Lichtman J. W. (2020). Long-range optogenetic control of axon guidance overcomes developmental boundaries and defects. *Dev. Cell* 53:577. 10.1016/J.DEVCEL.2020.05.009 32516597 PMC7375170

[B107] Harty B. L. Coelho F. Pease-Raissi S. E. Mogha A. Ackerman S. D. Herbert A. L. (2019). Myelinating Schwann cells ensheath multiple axons in the absence of E3 ligase component Fbxw7. *Nat. Commun.* 10:2976. 10.1038/s41467-019-10881-y 31278268 PMC6611888

[B108] Haynes S. E. Hollopeter G. Yang G. Kurpius D. Dailey M. E. Gan W. B. (2006). The P2Y12 receptor regulates microglial activation by extracellular nucleotides. *Nat. Neurosci.* 9 1512–1519. 10.1038/nn1805 17115040

[B109] Henke K. Bowen M. E. Harris M. P. (2013). Identification of mutations in zebrafish using next-generation sequencing. *Curr. Protoc. Mol. Biol.* 104 1–33. 10.1002/0471142727.mb0713s104 24510885

[B110] Herbert A. L. Fu M. Drerup C. M. Gray R. S. Harty B. L. Ackerman S. D. (2017). Dynein/dynactin is necessary for anterograde transport of Mbp mRNA in oligodendrocytes and for myelination in vivo. *Proc. Natl. Acad. Sci. U.S.A.* 114 9153–9162. 10.1073/PNAS.1711088114 29073112 PMC5664533

[B111] Herbomel P. (1999). Ontogeny and behaviour of early macrophages in the zebrafish embryo. *Curr. Biol.* 9 3735–3745. 10.1016/S0960-9822(99)80407-2 10433904

[B112] Herbomel P. Thisse B. Thisse C. (2001). Zebrafish early macrophages colonize cephalic mesenchyme and developing brain, retina, and epidermis through a M-CSF receptor-dependent invasive process. *Dev. Biol.* 238 274–288. 10.1006/dbio.2001.0393 11784010

[B113] Hill R. A. Li A. M. Grutzendler J. (2018). Lifelong cortical myelin plasticity and age-related degeneration in the live mammalian brain. *Nat. Neurosci.* 21 683–695. 10.1038/s41593-018-0120-6 29556031 PMC5920745

[B114] Hines J. H. Ravanelli A. M. Schwindt R. Scott E. K. Appel B. (2015). Neuronal activity biases axon selection for myelination in vivo. *Nat Neurosci* 18 683–689. 10.1038/nn.3992 25849987 PMC4414883

[B115] Holler K. Neuschulz A. Drewe-Boß P. Mintcheva J. Spanjaard B. Arsiè R. (2021). Spatio-temporal mRNA tracking in the early zebrafish embryo. *Nat. Commun.* 12 1–13. 10.1038/S41467-021-23834-1 34099733 PMC8184788

[B116] Hong S. Beja-glasser V. F. Nfonoyim B. M. Frouin A. Ramakrishnan S. Merry K. M. (2016). Complement and microglia mediate early synapse loss in alzheimer mouse models. *Curr. Alzheimer Res.* 352 712–716. 10.1126/science.aad8373.Complement PMC5094372 27033548

[B117] Howe K. Clark M. D. Torroja C. F. Torrance J. Berthelot C. Muffato M. (2013). The zebrafish reference genome sequence and its relationship to the human genome. *Nature* 496 498–503. 10.1038/nature12111 23594743 PMC3703927

[B118] Huang P. Zhu Z. Lin S. Zhang B. (2012). Reverse genetic approaches in zebrafish. *J. Genet. Genomics* 39 421–433. 10.1016/j.jgg.2012.07.004 23021542

[B119] Hudish L. I. Blasky A. J. Appel B. (2013). miR-219 regulates neural precursor differentiation by direct inhibition of apical par polarity proteins. *Dev. Cell* 27 387–398. 10.1016/J.DEVCEL.2013.10.015 24239515 PMC3862977

[B120] Hughes A. N. Appel B. (2019). Oligodendrocytes express synaptic proteins that modulate myelin sheath formation. *Nat. Commun.* 10:4125. 10.1038/s41467-019-12059-y 31511515 PMC6739339

[B121] Hughes A. N. Appel B. (2020). Microglia phagocytose myelin sheaths to modify developmental myelination. *Nat. Neurosci.* 23 1055–1066. 10.1038/s41593-020-0654-2 32632287 PMC7483351

[B401] Hughes E. G. Kang S. H. Fukaya M. Bergles D. E. (2013). Oligodendrocyte progenitors balance growth with self-repulsion to achieve homeostasis in the adult brain. *Nat. Neurosci.* 16, 668–679. 10.1038/NN.3390 23624515 PMC3807738

[B122] Hughes E. G. Orthmann-Murphy J. L. Langseth A. J. Bergles D. E. (2018). Myelin remodeling through experience-dependent oligodendrogenesis in the adult somatosensory cortex. *Nat. Neurosci.* 21 696–708. 10.1038/s41593-018-0121-5 29556025 PMC5920726

[B123] Jadhav A. P. Roesch K. Cepko C. L. (2009). Development and neurogenic potential of *Müller glial* cells in the vertebrate retina. *Prog. Retin. Eye Res.* 28 249–262. 10.1016/j.preteyeres.2009.05.002 19465144 PMC3233204

[B124] Jahn O. Siems S. B. Kusch K. Hesse D. Jung R. B. Liepold T. (2020). The CNS myelin proteome: deep profile and persistence after post-mortem delay. *Front. Cell. Neurosci.* 14:239. 10.3389/FNCEL.2020.00239 32973451 PMC7466725

[B125] Jahn O. Tenzer S. Werner H. B. (2009). Myelin proteomics: molecular anatomy of an insulating sheath. *Mol. Neurobiol.* 40 55–72. 10.1007/s12035-009-8071-2 19452287 PMC2758371

[B126] Jäkel S. Dimou L. (2017). Glial cells and their function in the adult brain: a journey through the history of their ablation. *Front. Cell. Neurosci.* 11:24. 10.3389/fncel.2017.00024 28243193 PMC5303749

[B127] James O. G. Selvaraj B. T. Magnani D. Burr K. Connick P. Barton S. K. (2021). iPSC-derived myelinoids to study myelin biology of humans. *Dev. Cell* 56 1346–1358. 10.1016/j.devcel.2021.04.006 33945785 PMC8098746

[B128] Jing X. Malicki J. (2009). Zebrafish ale oko, an essential determinant of sensory neuron survival and the polarity of retinal radial glia, encodes the p50 subunit of dynactin. *Development* 136 2955–2964. 10.1242/DEV.037739 19666822 PMC2723067

[B129] Johnson K. Moriarty C. Tania N. Ortman A. DiPietrantonio K. Edens B. (2014). Kif11 dependent cell cycle progression in radial glial cells is required for proper neurogenesis in the zebrafish neural tube. *Dev. Biol.* 387 73–92. 10.1016/j.ydbio.2013.12.021 24370453 PMC3936480

[B130] Jung S. Kim S. Chung A. Kim H. So J. Ryu J. (2010). Visualization of myelination in GFP-Transgenic zebrafish. *Dev. Dyn.* 239 592–597. 10.1002/dvdy.22166 19918882

[B131] Jurisch-yaksi N. (2020). Radial glia in the zebrafish brain?: functional, structural, and physiological comparison with the mammalian glia. *Glia* 68 2451–2470. 32476207 10.1002/glia.23849

[B132] Jurisch-Yaksi N. Yaksi E. Kizil C. (2020). Radial glia in the zebrafish brain: Functional, structural, and physiological comparison with the mammalian glia. *Glia* 68 2451–2470. 10.1002/glia.23849 32476207

[B133] Karttunen M. J. Czopka T. Goedhart M. Early J. J. Lyons D. A. De Castro F. (2017). Regeneration of myelin sheaths of normal length and thickness in the zebrafish CNS correlates with growth of axons in caliber. *PLoS One* 12:e0178058. 10.1371/journal.pone.0178058 28542521 PMC5444792

[B134] Kato D. Wake H. Lee P. R. Tachibana Y. Ono R. Sugio S. (2019). Motor learning requires myelination to reduce asynchrony and spontaneity in neural activity. *Glia* 68 1–18. 10.1002/glia.23713 31465122 PMC6899965

[B135] Kawakami K. Takeda H. Kawakami N. Kobayashi M. Matsuda N. Mishina M. (2004). A transposon-mediated gene trap approach identifies developmentally regulated genes in zebrafish. *Dev. Cell* 7 133–144. 10.1016/j.devcel.2004.06.005 15239961

[B136] Kazakova N. Li H. Mora A. Jessen K. R. Mirsky R. Richardson W. D. (2006). A screen for mutations in zebrafish that affect myelin gene expression in Schwann cells and oligodendrocytes. *Dev. Biol.* 297 1–13. 10.1016/j.ydbio.2006.03.020 16839543

[B137] Kearns C. A. Ravanelli A. M. Cooper K. Appel B. (2015). Fbxw7 limits myelination by inhibiting mtor signaling. *J. Neurosci.* 35 14861–14871. 10.1523/JNEUROSCI.4968-14.2015 26538655 PMC4635133

[B138] Kearns C. A. Walker M. Ravanelli A. M. Scott K. Arzbecker M. R. Appel B. (2021). Zebrafish spinal cord oligodendrocyte formation requires boc function. *Genetics* 218, 1–13. 10.1093/genetics/iyab082 34057474 PMC8864740

[B139] Keatinge M. Tsarouchas T. M. Munir T. Porter N. J. Larraz J. Gianni D. (2021). CRISPR gRNA phenotypic screening in zebrafish reveals pro-regenerative genes in spinal cord injury. *PLoS Genet.* 17:e1009515. 10.1371/journal.pgen.1009515 33914736 PMC8084196

[B140] Kegel L. Rubio M. Almeida R. G. Benito S. Klingseisen A. Lyons D. A. (2019). “Forward genetic screen using zebrafish to identify new genes involved in myelination,” in *Oligodendrocytes: Methods and Protocols*, eds Lyons D. A. Kegel L. (New York, NY: Springer New York), 185–209. 10.1007/978-1-4939-9072-6_11 30820900

[B141] Kim D. H. Kim J. Marques J. C. Grama A. Hildebrand D. G. C. Gu W. (2017). Pan-neuronal calcium imaging with cellular resolution in freely swimming zebrafish. *Nat. Methods* 14 1107–1114. 10.1038/nmeth.4429 28892088

[B142] Kim H. Shin J. Kim S. Poling J. Park H.-C. Appel B. (2008). Notch-regulated oligodendrocyte specification from radial glia in the spinal cord of zebrafish embryos. *Dev. Dyn.* 237: 2081. 10.1002/DVDY.21620 18627107 PMC2646814

[B143] Kirby B. B. Takada N. Latimer A. J. Shin J. Carney T. J. Kelsh R. N. (2006). In vivo time-lapse imaging shows dynamic oligodendrocyte progenitor behavior during zebrafish development. *Nat. Neurosci.* 9 1506–1511. 10.1038/nn1803 17099706

[B144] Klatt Shaw D. Mokalled M. H. (2021). Efficient CRISPR/Cas9 mutagenesis for neurobehavioral screening in adult zebrafish. *G3 Genes Genomes Genet.* 10.1093/g3journal/jkab089 33742663 PMC8496216

[B145] Klingseisen A. Lyons D. A. (2018). Axonal regulation of central nervous system myelination: structure and function. *Neuroscientist* 24 7–21. 10.1177/1073858417703030 28397586

[B146] Klingseisen A. Ristoiu A. Kegel L. Poole R. J. Brophy P. J. Lyons D. A. (2019). Oligodendrocyte neurofascin independently regulates both myelin targeting and sheath growth in the CNS article oligodendrocyte neurofascin independently regulates both myelin targeting and sheath growth in the CNS. *Dev. Cell* 51 730–744.e6. 10.1016/j.devcel.2019.10.016 31761670 PMC6912162

[B147] Koudelka S. Voas M. G. G. Almeida R. G. G. Baraban M. Soetaert J. Meyer M. P. P. (2016). Individual neuronal subtypes exhibit diversity in CNS myelination mediated by synaptic vesicle release. *Curr. Biol.* 26 1447–1455. 10.1016/j.cub.2016.03.070 27161502 PMC4906267

[B148] Krasnow A. M. Ford M. C. Valdivia L. E. Wilson S. W. (2018). Europe PMC Funders Group Regulation of developing myelin sheath elongation by oligodendrocyte calcium transients in vivo. *Nat. Neurosci.* 21 24–28. 10.1038/s41593-017-0031-y.Regulation 29230052 PMC6478117

[B149] Kriegstein A. R. Götz M. (2003). Radial glia diversity: a matter of cell fate. *Glia* 43 37–43. 10.1002/GLIA.10250 12761864

[B150] Kroehne V. Freudenreich D. Hans S. Kaslin J. Brand M. (2011). Regeneration of the adult zebrafish brain from neurogenic radial glia-type progenitors. *Development* 138 4831–4841. 10.1242/dev.072587 22007133

[B151] Kroll F. Powell G. T. Ghosh M. Gestri G. Antinucci P. Hearn T. J. (2021). A simple and effective f0 knockout method for rapid screening of behaviour and other complex phenotypes. *elife* 10:e59683. 10.7554/eLife.59683 33416493 PMC7793621

[B152] Kucenas S. (2015). Perineurial glia. *Cold Spring Harb. Perspect. Biol.* 7 1–14. 10.1101/cshperspect.a020511 25818566 PMC4448606

[B153] Kucenas S. Snell H. Appel B. (2008). Nkx2.2a promotes specification and differentiation of a myelinating subset of oligodendrocyte lineage cells in zebrafish. *Neuron Glia Biol.* 4 71–81. 10.1017/S1740925X09990123 19737431 PMC2821284

[B154] Kuil L. E. López Martí A. Carreras Mascaro A. van den Bosch J. C. van den Berg P. van der Linde H. C. (2019a). Hexb enzyme deficiency leads to lysosomal abnormalities in radial glia and microglia in zebrafish brain development. *Glia* 67 1705–1718. 10.1002/glia.23641 31140649 PMC6772114

[B155] Kuil L. E. Oosterhof N. Geurts S. N. Van Der Linde H. C. Meijering E. Van Ham T. J. (2019b). Reverse genetic screen reveals that Il34 facilitates yolk sac macrophage distribution and seeding of the brain. *DMM Dis. Model. Mech.* 12 1–12. 10.1242/dmm.037762 30765415 PMC6451432

[B156] Kyritsis N. Kizil C. Zocher S. Kroehne V. Kaslin J. Freudenreich D. (2012). Acute inflammation initiates the regenerative response in the adult zebrafish brain. *Science* 338 1353–1356. 10.1126/science.1228773 23138980

[B157] Lamason R. L. Mohideen M. P. K. Mest J. R. Wong A. C. Norton H. L. Aros M. C. (2005). SLC24A5, a putative cation exchanger, affects pigmentation in zebrafish and humans. *Science* 310 1782–1787. 16357253 10.1126/science.1116238

[B158] Lawson N. D. Wolfe S. A. (2011). Perspective forward and reverse genetic approaches for the analysis of vertebrate development in the zebrafish. *Dev. Cell* 21 48–64. 10.1016/j.devcel.2011.06.007 21763608

[B159] Lee C. Lavoie A. Liu J. Chen S. X. Liu B. Chen S. X. (2020). Light up the brain?: the application of optogenetics in cell-type specific dissection of mouse brain circuits. *Front. Neural Circuits* 14:18. 10.3389/fncir.2020.00018 32390806 PMC7193678

[B160] Lee J.-S. Padmanabhan A. Shin J. Zhu S. Guo F. Kanki J. P. (2010). Oligodendrocyte progenitor cell numbers and migration are regulated by the zebrafish orthologs of the NF1 tumor suppressor gene. *Hum. Mol. Genet.* 19 4643–4653. 10.1093/HMG/DDQ395 20858602 PMC3999377

[B161] Lee S. Leach M. K. Redmond S. A. Chong S. Y. C. Mellon S. H. Tuck S. J. (2012). A culture system to study oligodendrocyte myelination processes using engineered nanofibers. *Nat. Methods* 9 917–922. 10.1038/nmeth.2105 22796663 PMC3433633

[B162] Lee S. Park J. Lee W. Kim H. Park H. Mori K. (2009). Lipocalin-2 is an autocrine mediator of reactive astrocytosis. *J. Neurosci.* 29 234–249. 10.1523/JNEUROSCI.5273-08.2009 19129400 PMC6664907

[B163] Li H. Lu Y. Smith H. K. Richardson W. D. (2007). Olig1 and Sox10 interact synergistically to drive myelin basic protein transcription in oligodendrocytes. *J. Neurosci.* 27 14375–14382. 10.1523/JNEUROSCI.4456-07.2007 18160645 PMC6329447

[B164] Li L. Jin H. Xu J. Shi Y. Wen Z. (2011). Irf8 regulates macrophage versus neutrophil fate during zebrafish primitive myelopoiesis. *Blood* 117 1359–1369. 10.1182/blood-2010-06-290700 21079149

[B165] Li Y. Du X. F. Liu C. S. Wen Z. L. Du J. L. (2012). Reciprocal regulation between resting microglial dynamics and neuronal activity in vivo. *Dev. Cell* 23 1189–1202. 10.1016/j.devcel.2012.10.027 23201120

[B166] Lieschke G. J. Currie P. D. (2010). Animal models of human disease?: zebrafish swim into view. *Nat. Rev. Genet.* 8 353–367. 10.1038/nrg2091 17440532

[B167] Lieschke G. J. Oates A. C. Paw B. H. Thompson M. A. Hall N. E. Ward A. C. (2002). Zebrafish SPI-1 (PU.1) marks a site of myeloid development independent of primitive erythropoiesis: implications for axial patterning. *Dev. Biol.* 246 274–295. 10.1006/dbio.2002.0657 12051816

[B168] Lister J. A. Robertson C. P. Lepage T. Johnson S. L. Raible D. W. (1999). Nacre encodes a zebrafish microphthalmia-related protein that regulates neural-crest-derived pigment cell fate. *Development* 126 3757–3767. 10.1242/dev.126.17.3757 10433906

[B169] Liu J. Zhou Y. Qi X. Chen J. Chen W. Qiu G. (2017). CRISPR/Cas9 in zebrafish: an efficient combination for human genetic diseases modeling. *Hum. Genet.* 136 1–12. 10.1007/s00439-016-1739-6 27807677 PMC5214880

[B170] Liu K. Petree C. Requena T. Varshney P. Varshney G. K. (2019). Expanding the CRISPR toolbox in zebrafish for studying development and disease. *Front. Cell Dev. Biol.* 7:13. 10.3389/FCELL.2019.00013 30886848 PMC6409501

[B171] Lu Q. R. Sun T. Zhu Z. Ma N. Garcia M. Stiles C. D. (2002). Common developmental requirement for Olig function indicates a motor neuron/oligodendrocyte connection. *Cell* 109 75–86. 10.1016/S0092-8674(02)00678-5 11955448

[B172] Lu Q. R. Yuk D. I. Alberta J. A. Zhu Z. Pawlitzky I. Chan J. (2000). Sonic hedgehog-regulated oligodendrocyte lineage genes encoding bHLH proteins in the mammalian central nervous system. *Neuron* 25 317–329. 10.1016/S0896-6273(00)80897-1 10719888

[B173] Lyons D. A. Kegel L. (2019). *Oligodendrocytes Methods and Protocols Methods in Molecular Biology 1936.* Available online at: http://www.springer.com/series/7651 (accessed July, 2019).

[B174] Lyons D. A. Talbot W. S. (2015). Glial cell development and function in zebrafish. *Cold Spring Harb. Perspect. Biol.* 7 1–21. 10.1101/cshperspect.a020586 25395296 PMC4315925

[B175] Lyons D. A. Naylor S. G. Scholze A. Talbot W. S. (2009). Kif1b is essential for mRNA localization in oligodendrocytes and development of myelinated axons. *Nat. Genet.* 41 854–858. 10.1038/ng.376 19503091 PMC2702462

[B176] Ma D. Liu F. (2015). Genome editing and its applications in model organisms. *Genomics Proteomics Bioinformatics* 13 336–344. 10.1016/j.gpb.2015.12.001 26762955 PMC4747648

[B177] MacDonald R. B. Randlett O. Oswald J. Yoshimatsu T. Franze K. Harris W. A. (2015). Müller glia provide essential tensile strength to the developing retina. *J. Cell Biol.* 210 1075–1083. 10.1083/JCB.201503115 26416961 PMC4586739

[B178] Macrae C. A. Peterson R. T. (2015). Zebrafish as tools for drug discovery. *Nat. Publ. Gr.* 14 721–731. 10.1038/nrd4627 26361349

[B179] Madden M. E. Suminaite D. Ortiz E. Early J. E. Koudelka S. Livesey M. R. (2021). Central nervous system hypomyelination disrupts axonal conduction and behaviour in larval zebrafish. *J. Neurosci.* 41, 9099–9111. 10.1523/JNEUROSCI.0842-21.2021 34544838 PMC8570833

[B180] Marisca R. Hoche T. Agirre E. Hoodless L. J. Barkey W. Auer F. (2020). Functionally distinct subgroups of oligodendrocyte precursor cells integrate neural activity and execute myelin formation. *Nat. Neurosci.* 23 363–374. 10.1038/s41593-019-0581-2 32066987 PMC7292734

[B181] Marshall-Phelps K. L. H. Kegel L. Baraban M. Ruhwedel T. Almeida R. G. Rubio-Brotons M. (2020). Neuronal activity disrupts myelinated axon integrity in the absence of NKCC1b. *J. Cell Biol.* 219 1–12. 10.1083/JCB.201909022 32364583 PMC7337504

[B182] Mathews E. S. Appel B. (2016b). *Oligodendrocyte Differentiation.* Amsterdam: Elsevier Ltd, 10.1016/bs.mcb.2015.12.004 27312491

[B183] Mathews E. S. Appel B. (2016a). Cholesterol biosynthesis supports myelin gene expression and axon ensheathment through modulation of P13K/Akt/mTor signaling. *J. Neurosci.* 36 7628–7639. 10.1523/JNEUROSCI.0726-16.2016 27445141 PMC4951573

[B184] Mathews E. S. Mawdsley D. J. Walker M. Hines J. H. Pozzoli M. Appel B. (2014). Mutation of 3-hydroxy-3-methylglutaryl CoA synthase I reveals requirements for isoprenoid and cholesterol synthesis in oligodendrocyte migration arrest, axon wrapping, and myelin gene expression. *J. Neurosci.* 34 3402–3412. 10.1523/JNEUROSCI.4587-13.2014 24573296 PMC3935092

[B185] Mazaheri F. Breus O. Durdu S. Haas P. Wittbrodt J. Gilmour D. (2014). Distinct roles for BAI1 and TIM-4 in the engulfment of dying neurons by microglia. *Nat. Commun.* 5 1–11. 10.1038/ncomms5046 24898390

[B186] Mazzolini J. Le Clerc S. Morisse G. Coulonges C. Kuil L. E. Ham T. J. (2019). Gene expression profiling reveals a conserved microglia signature in larval zebrafish. *Glia* 68 1–18. 10.1002/glia.23717 31508850 PMC6916425

[B187] Mcclenahan P. Troup M. Scott E. K. (2012). Fin-Tail coordination during escape and predatory behavior in larval zebrafish. *PLoS One* 7:e32295. 10.1371/journal.pone.0032295 22359680 PMC3281131

[B188] McKeown K. A. Moreno R. Hall V. L. Ribera A. B. Downes G. B. (2012). Disruption of Eaat2b, a glutamate transporter, results in abnormal motor behaviors in developing zebrafish. *Dev. Biol.* 362 162–171. 10.1016/J.YDBIO.2011.11.001 22094018 PMC4013685

[B189] Meireles A. M. Shen K. Zoupi L. Iyer H. Bouchard E. L. Williams A. (2018). The lysosomal transcription factor tfeb represses myelination downstream of the rag-ragulator complex developmental cell the lysosomal transcription factor TFEB represses myelination downstream of the rag-ragulator complex. *Dev. Cell* 47 319–330. 10.1016/j.devcel.2018.10.003 30399334 PMC6250074

[B190] Meireles A. M. Shiau C. E. Kingsley D. M. Talbot W. S. Meireles A. M. Shiau C. E. (2014). The phosphate exporter xpr1b is required for differentiation of tissue-resident macrophages report the phosphate exporter xpr1b is required for differentiation of tissue-resident macrophages. *Cell Rep.* 8 1659–1667. 10.1016/j.celrep.2014.08.018 25220463 PMC4177277

[B191] Mensch S. Baraban M. Almeida R. Czopka T. Ausborn J. El Manira A. (2015). Synaptic vesicle release regulates the number of myelin sheaths made by individual oligodendrocytes in vivo. *Nat. Neurosci.* 18 628–630. 10.1038/nn.3991.Synaptic 25849985 PMC4427868

[B192] Mitew S. Hay C. M. Peckham H. Xiao J. Koenning M. Emery B. (2014). Mechanisms regulating the development of oligodendrocytes and central nervous system myelin. *Neuroscience* 276 29–47. 10.1016/j.neuroscience.2013.11.029 24275321

[B193] Mokalled M. H. Patra C. Dickson A. L. Endo T. Stainier D. Y. R. Poss K. D. (2016). Injury-induced ctgfa directs glial bridging and spinal cord regeneration in zebrafish. *Science* 354 630–634. 10.1126/science.aaf2679 27811277 PMC5114142

[B194] Monje M. (2018). Myelin plasticity and nervous system function. *Annu. Rev. Neurosci.* 41 61–76. 10.1146/annurev-neuro-080317-061853 29986163

[B195] Monk K. R. Feltri M. L. Taveggia C. (2015). New insights on schwann cell development. *Glia* 63 1376–1393. 10.1002/glia.22852.New 25921593 PMC4470834

[B196] Monk K. R. Naylor S. G. Glenn T. D. Mercurio S. Perlin J. R. Dominguez C. (2009). A G protein-coupled receptor is essential for schwann cells to initiate myelination. *Science* 325 1402–1405. 10.1126/science.1173474 19745155 PMC2856697

[B197] Moyon S. Frawley R. Marechal D. Huang D. Marshall-Phelps K. L. H. Kegel L. (2021). TET1-mediated DNA hydroxymethylation regulates adult remyelination in mice. *Nat. Commun.* 12:3359. 10.1038/s41467-021-23735-3 34099715 PMC8185117

[B198] Mruk K. Ciepla P. Piza P. A. Alnaqib M. A. Chen J. K. (2020). Targeted cell ablation in zebrafish using optogenetic transcriptional control. *Development* 147 1–11. 10.1242/dev.183640 32414936 PMC7328002

[B199] Mu Y. Bennett D. V. Rubinov M. Narayan S. Yang C. T. Tanimoto M. (2019). Glia accumulate evidence that actions are futile and suppress unsuccessful behavior. *Cell* 178 27–43.e19. 10.1016/j.cell.2019.05.050 31230713

[B200] Muppirala A. N. Limbach L. E. Bradford E. F. Petersen S. C. (2020). Schwann cell development: From neural crest to myelin sheath. *Wiley Interdiscip. Rev. Dev. Biol.* 10, 1–34. 10.1002/wdev.398 33145925

[B201] Nasevicius A. Ekker S. C. (2000). Effective targeted gene “knockdown” in zebrafish. *Nat. Genet.* 26 216–220. 10.1038/79951 11017081

[B202] Nave K. A. (2010). Myelination and support of axonal integrity by glia. *Nature* 468 244–252. 10.1038/nature09614 21068833

[B203] Nave K. A. Ehrenreich H. (2018). A bloody brake on myelin repair. *Nature* 553 31–32. 10.1038/d41586-017-08232-2 32080637

[B204] Nave K.-A. Werner H. B. (2014). Myelination of the nervous system: mechanisms and functions. *Annu. Rev. Cell Dev. Biol.* 30 503–533. 10.1146/annurev-cellbio-100913-013101 25288117

[B205] Nawaz S. Sánchez P. Schmitt S. Snaidero N. Mitkovski M. Velte C. (2015). Actin filament turnover drives leading edge growth during myelin sheath formation in the central nervous system. *Dev. Cell* 34 139–151. 10.1016/j.devcel.2015.05.013 26166299 PMC4736019

[B206] Neely S. A. Williamson J. M. Klingseisen A. Zoupi L. Early J. J. Williams A. (2020). New oligodendrocytes exhibit more abundant and accurate myelin regeneration than those that survive demyelination. *bioRxiv* [Preprint] 10.1101/2020.05.22.110551 PMC7612594 35165460

[B207] Nelson C. M. Ackerman K. M. Hayer P. O. Bailey T. J. Gorsuch R. A. Hyde D. R. (2013). Tumor necrosis factor-alpha is produced by dying retinal neurons and is required for müller glia proliferation during zebrafish retinal regeneration. *J. Neurosci.* 33 6524–6539. 10.1523/JNEUROSCI.3838-12.2013 23575850 PMC3740543

[B208] Nelson H. N. Treichel A. J. Eggum E. N. Martell M. R. Kaiser A. J. Trudel A. G. (2020). Individual neuronal subtypes control initial myelin sheath growth and stabilization. *Neural Dev.* 15 1–17. 10.1186/s13064-020-00149-3 32988384 PMC7523326

[B209] Nett W. J. Oloff S. H. Mccarthy K. D. (2002). Hippocampal astrocytes in situ exhibit calcium oscillations that occur independent of neuronal activity. *J. Neurophysiol.* 87 528–537. 10.1152/jn.00268.2001 11784768

[B210] Nijhawan D. Honarpour N. Wang X. (2000). Apoptosis in neural development and disease. *Annu. Rev. Neurosci.* 23 73–87. 10845059 10.1146/annurev.neuro.23.1.73

[B211] Niklaus S. Cadetti L. Berg-maurer C. M. Lehnherr A. Adriana L. Forster I. C. (2017). Shaping of signal transmission at the photoreceptor synapse by EAAT2 glutamate transporters. *eNeuro* 4 1–17. 10.1523/ENEURO.0339-16.2017 PMC5467398 28612046

[B212] Norenberg M. D. Martinez-Hernandez A. (1979). Fine structural localization of glutamine synthetase in astrocytes of rat brain. *Brain Res.* 161 303–310. 31966 10.1016/0006-8993(79)90071-4

[B213] Nüsslein-Volhard C. N. Wieschaus E. (1980). Mutations affecting segment number and polarity in *Drosophila* christiane. *Nature* 287 797–801. 10.1038/287795a0 6776413

[B214] Nutma E. van Gent D. Amor S. Peferoen L. A. N. (2020). astrocyte and oligodendrocyte cross-talk in the central nervous system. *Cells* 9 1–21. 10.3390/cells9030600 32138223 PMC7140446

[B215] Oberheim N. A. Goldman S. A. Nedergaard M. (2012). Heterogeneity of astrocytic form and function. *Methods Mol. Biol.* 814 23–45. 10.1007/978-1-61779-452-0_3 22144298 PMC3506190

[B216] Okochi Y. Sakaguchi S. Nakae K. Kondo T. Naoki H. (2021). Model-based prediction of spatial gene expression via generative linear mapping. *Nat. Commun.* 12 1–13. 10.1038/S41467-021-24014-X 34140477 PMC8211835

[B217] Oosterhof N. Kuil L. E. van der Linde H. C. Burm S. M. Berdowski W. van Ijcken W. F. J. (2018). Colony-stimulating factor 1 receptor (CSF1R) regulates microglia density and distribution, but not microglia differentiation in vivo. *Cell Rep* 24 1203–1217.e6. 10.1016/j.celrep.2018.06.113 30067976

[B218] Orger M. B. De Polavieja G. G. (2017). Zebrafish behavior?: opportunities and challenges. *Annu. Rev. Neurosci.* 40 125–147. 28375767 10.1146/annurev-neuro-071714-033857

[B219] Orger M. B. Smear M. C. Anstis S. M. Baier H. (2000). Perception of fourier and non-fourier motion by larval zebrafish. *Nat. Neurosci.* 3 1128–1133. 10.1038/80649 11036270

[B220] Papadopoulos M. C. Verkman A. S. (2013). Aquaporin water channels in the nervous system. *Nat. Rev. Neurosci.* 14 265–277. 10.1038/nrn3468 23481483 PMC3732112

[B221] Park H. C. Mehta A. Richardson J. S. Appel B. (2002). Olig2 is required for zebrafish primary motor neuron and oligodendrocyte development. *Dev. Biol.* 248 356–368. 10.1006/dbio.2002.0738 12167410

[B222] Park H. Appel B. (2003). Delta-notch signaling regulates oligodendrocyte specification. *Development* 130 3747–3755. 10.1242/dev.00576 12835391

[B223] Park H. Shin J. Appel B. (2004). Spatial and temporal regulation of ventral spinal cord precursor specification by Hedgehog signaling. *Development* 131 5959–5969. 10.1242/dev.01456 15539490

[B224] Patton E. E. Zon L. I. (2001). The art and design of genetic screens: zebrafish. *Nat. Rev. Genet.* 2 956–966. 11733748 10.1038/35103567

[B225] Patton E. E. Zon L. I. Langenau D. M. (2021). Zebrafish disease models in drug discovery: from preclinical modelling to clinical trials. *Nat. Rev. Drug Discov.* 20 611–628. 10.1038/s41573-021-00210-8 34117457 PMC9210578

[B226] Paukert M. Agarwal A. Cha J. Doze V. A. Kang J. U. Bergles D. E. (2014). Norepinephrine controls astroglial responsiveness to local circuit activity. *Neuron* 82 1263–1270. 10.1016/j.neuron.2014.04.038 24945771 PMC4080721

[B227] Perez-Catalan N. A. Doe C. Q. Ackerman S. D. (2021). The role of astrocyte−mediated plasticity in neural circuit development and function. *Neural Dev.* 16:1. 10.1186/S13064-020-00151-9 33413602 PMC7789420

[B228] Peri F. Nüsslein-Volhard C. (2008). Live imaging of neuronal degradation by microglia reveals a role for v0-atpase a1 in phagosomal fusion in vivo. *Cell* 133 916–927. 10.1016/j.cell.2008.04.037 18510934

[B229] Perlin J. R. Lush M. E. Zac Stephens W. Piotrowski T. Talbot W. S. (2011). Neuronal neuregulin 1 type III directs schwann cell migration. *Development* 138 4639–4648. 10.1242/dev.068072 21965611 PMC3190382

[B230] Piller M. Werkman I. L. Brown E. A. Latimer A. J. Kucenas S. (2021). Glutamate signaling via the AMPAR subunit GluR4 regulates oligodendrocyte progenitor cell migration in the developing spinal cord. *J. Neurosci.* 41 5353–5371. 10.1523/jneurosci.2562-20.2021 33975920 PMC8221590

[B231] Pogoda H. M. Sternheim N. Lyons D. A. Diamond B. Hawkins T. A. Woods I. G. (2006). A genetic screen identifies genes essential for development of myelinated axons in zebrafish. *Dev. Biol.* 298 118–131. 10.1016/j.ydbio.2006.06.021 16875686

[B232] Poskanzer K. E. Molofsky A. V. (2018). Dynamism of an astrocyte in vivo: perspectives on identity and function. *Annu. Rev. Physiol.* 10 143–157. 10.1146/annurev-physiol-021317-121125 29166242 PMC5811396

[B233] Powell C. Cornblath E. Elsaeidi F. Wan J. Goldman D. (2016). Zebrafish Müller glia-derived progenitors are multipotent, exhibit proliferative biases and regenerate excess neurons. *Sci. Rep.* 6 1–10. 10.1038/srep24851 27094545 PMC4837407

[B234] Preston M. A. Macklin W. B. (2015). Zebrafish as a model to investigate CNS myelination. *Glia* 63 177–193. 10.1002/glia.22755 25263121 PMC4539269

[B235] Preston M. A. Finseth L. T. Bourne J. N. Macklin W. B. (2019). A novel myelin protein zero transgenic zebrafish designed for rapid readout of in vivo myelination. *Glia* 67 650–667. 10.1002/glia.23559 30623975 PMC6555554

[B236] Ransohoff R. M. Cardona A. E. (2010). The myeloid cells of the central nervous system parenchyma. *Nature* 468 253–262. 10.1038/nature09615 21068834

[B237] Ravanelli A. M. Appel B. (2015). Motor neurons and oligodendrocytes arise from distinct cell lineages by progenitor recruitment. *Genes Dev.* 29 2504–2515. 10.1101/gad.271312.115 26584621 PMC4691953

[B238] Ravanelli A. M. Kearns C. A. Powers R. K. Wang Y. Hines J. H. Donaldson M. J. (2018). Sequential specification of oligodendrocyte lineage cells by distinct levels of Hedgehog and Notch signaling. *Dev. Biol.* 444 93–106. 10.1016/j.ydbio.2018.10.004 30347186 PMC6263812

[B239] Rhodes J. Hagen A. Hsu K. Deng M. Liu T. X. Look A. T. (2005). Interplay of pu.1 and Gata1 determines myelo-erythroid progenitor cell fate in zebrafish. *Dev. Cell* 8 97–108. 10.1016/j.devcel.2004.11.014 15621533

[B240] Richardson W. D. Kessaris N. Pringle N. (2006). Oligodendrocyte wars. *Nat. Rev. Neurosci.* 7 11–18. 10.1038/nrn1826 16371946 PMC6328010

[B241] Rossi F. Casano A. M. Henke K. Richter K. Peri F. (2015). The SLC7A7 transporter identifies microglial precursors prior to entry into the brain. *Cell Rep.* 11 1008–1017. 10.1016/j.celrep.2015.04.028 25959825

[B242] Sakry D. Neitz A. Singh J. Frischknecht R. Marongiu D. Binamé F. (2014). Oligodendrocyte precursor cells modulate the neuronal network by activity-dependent ectodomain cleavage of Glial NG2. *PLoS Biol.* 12:e1001993. 10.1371/journal.pbio.1001993 25387269 PMC4227637

[B243] Saleem S. Kannan R. R. (2018). Zebrafish: an emerging real-time model system to study Alzheimer’s disease and neurospecific drug discovery. *Cell Death Discov.* 4 1–13. 10.1038/s41420-018-0109-7 30302279 PMC6170431

[B244] Sancho L. Contreras M. Allen N. J. (2021). Glia as sculptors of synaptic plasticity. *Neurosci. Res.* 167 17–29. 10.1016/J.NEURES.2020.11.005 33316304 PMC8513541

[B245] Schirmer L. Schafer D. P. Bartels T. Rowitch D. H. Calabresi P. A. (2021). Diversity and function of glial cell types in multiple sclerosis. *Trends Immunol.* 42 228–247. 10.1016/j.it.2021.01.005 33593693 PMC7914214

[B246] Schulz C. Perdiguero E. G. Chorro L. Szabo-Rogers H. Cagnard N. Kierdorf K. (2012). A lineage of myeloid cells independent of myb and hematopoietic stem cells. *Science* 335 86–90. 10.1126/science.1219179 22442384

[B247] Scott K. O’Rourke R. Gillen A. Appel B. (2020). Prdm8 regulates pMN progenitor specification for motor neuron and oligodendrocyte fates by modulating the Shh signaling response. *Development* 147 1–15. 10.1242/dev.191023 32680935 PMC7473643

[B248] Shaham S. (2015). Glial development and function in the nervous system of *Caenorhabditis elegans*. *Cold Spring Harb. Perspect. Biol.* 7 1–14. 10.1101/cshperspect.a020578 25573712 PMC4382739

[B249] Shen K. Sidik H. Talbot W. S. (2016). The Rag-ragulator complex regulates lysosome function and phagocytic flux in microglia. *Cell Rep.* 14 547–599. 26774477 10.1016/j.celrep.2015.12.055 PMC4731305

[B250] Sherman D. L. Brophy P. J. (2005). Mechanisms of axon ensheathment and myelin growth. *Nat. Rev. Neurosci.* 6 683–690. 10.1038/nrn1743 16136172

[B251] Shiau C. E. Kaufman Z. Meireles A. M. Talbot W. S. (2015). Differential requirement for irf8 in formation of embryonic and adult macrophages in zebrafish. *PLoS One* 10:e0117513. 10.1371/journal.pone.0117513 25615614 PMC4304715

[B252] Shiau C. E. Monk K. R. Joo W. Talbot W. S. (2014). An anti-inflammatory NOD-like receptor is required for microglia development. *Cell Rep.* 5 1–21. 10.1016/j.celrep.2013.11.004.An PMC3878655 24316075

[B253] Shin J. Padmanabhan A. de Groh E. D. Lee J.-S. Haidar S. Dahlberg S. (2012). Zebrafish neurofibromatosis type 1 genes have redundant functions in tumorigenesis and embryonic development. *Dis. Model. Mech.* 5:881. 10.1242/DMM.009779 22773753 PMC3484870

[B254] Shin J. Park H. C. Topczewska J. M. Madwsley D. J. Appel B. (2003). Neural cell fate analysis in zebrafish using olig2 BAC transgenics. *Methods Cell Sci.* 25 7–14. 10.1023/B:MICS.0000006847.09037.3a 14739582

[B255] Sidik H. Talbot W. S. (2015). A zinc finger protein that regulates oligodendrocyte specification, migration and myelination in zebrafish. *Development* 142 4119–4128. 10.1242/DEV.128215 26459222 PMC4712842

[B256] Sieger D. Moritz C. Ziegenhals T. Prykhozhij S. Peri F. (2012). Article long-range Ca 2 + waves transmit brain-damage signals to microglia. *Dev. Cell* 22 1138–1148. 10.1016/j.devcel.2012.04.012 22632801

[B257] Siems S. B. Jahn O. Hoodless L. J. Jung R. B. Hesse D. Möbius W. (2021). Proteome profile of myelin in the zebrafish brain. *Front. Cell Dev. Biol.* 9: 640169. 10.3389/fcell.2021.640169 33898427 PMC8060510

[B258] Sifuentes C. J. Kim J. W. Swaroop A. Raymond P. A. (2016). Rapid, dynamic activation of *Müller Glial* stem cell responses in Zebrafish. *Investig. Ophthalmol. Vis. Sci.* 57 5148–5160. 10.1167/iovs.16-19973 27699411 PMC5054728

[B259] Silva N. J. Dorman L. C. Vainchtein I. D. Horneck N. C. Molofsky A. V. (2021). In situ and transcriptomic identification of synapse-associated microglia in the developing zebrafish brain. *bioRxiv* [Preprint] 10.1101/2021.05.08.443268 PMC8501082 34625548

[B260] Snaidero N. Möbius W. Czopka T. Hekking L. H. P. Mathisen C. Verkleij D. (2014). Myelin membrane wrapping of CNS axons by PI(3,4,5)P3-dependent polarized growth at the inner tongue. *Cell* 156 277–290. 10.1016/j.cell.2013.11.044 24439382 PMC4862569

[B261] Snyder J. L. Kearns C. A. Appel B. (2012). Fbxw7 regulates Notch to control specification of neural precursors for oligodendrocyte fate. *Neural Dev.* 7 1–12. 22554084 10.1186/1749-8104-7-15 PMC3404928

[B262] Somjen G. G. (1988). Nervenkitt: notes on the history of the concept of neuroglia. *Glia* 1 2–9. 10.1002/glia.440010103 2976736

[B263] Stadelmann C. Timmler S. Barrantes-Freer A. Simons M. (2019). Myelin in the central nervous system: structure, function, and pathology. *Physiol. Rev.* 99 1381–1431. 10.1152/physrev.00031.2018 31066630

[B264] Stainier D. Y. R. Raz E. Lawson N. D. Ekker S. C. Burdine R. D. Eisen J. S. (2017). Guidelines for morpholino use in zebrafish. *PLoS Genet.* 13:e1007000. 10.1371/journal.pgen.1007000 29049395 PMC5648102

[B265] Stassart R. M. Möbius W. Nave K. A. Edgar J. M. (2018). The axon-myelin unit in development and degenerative disease. *Front. Neurosci.* 12:467. 10.3389/fnins.2018.00467 30050403 PMC6050401

[B266] Stevens B. Allen N. J. Vazquez L. E. Howell G. R. Christopherson K. S. Nouri N. (2007). The classical complement cascade mediates CNS synapse elimination. *Cell* 131 1164–1178. 10.1016/j.cell.2007.10.036 18083105

[B267] Stewart A. M. Braubach O. Spitsbergen J. Gerlai R. Kalueff A. V. (2014). Zebrafish models for translational neuroscience research?: from tank to bedside. *Trends Neurosci.* 37 264–278. 10.1016/j.tins.2014.02.011 24726051 PMC4039217

[B268] Stogsdill J. A. Eroglu C. (2017). The interplay between neurons and glia in synapse development and plasticity. *Curr. Opin. Neurobiol.* 42 1–15. 10.1016/J.CONB.2016.09.016 27788368 PMC5316301

[B269] Stogsdill J. A. Ramirez J. Liu D. Kim Y. H. Baldwin K. T. Enustun E. (2017). Astrocytic neuroligins control astrocyte morphogenesis and synaptogenesis. *Nature* 551 192–197. 10.1038/nature24638 29120426 PMC5796651

[B270] Streisinger G. Walker C. Dower N. Knauber D. Singer F. (1981). Production of clones of homozygous diploid zebra fish (*Brachydanio rerio*). *Nature* 291 293–296. 7248006 10.1038/291293a0

[B271] Suminaite D. Lyons D. A. Livesey M. R. (2019). Myelinated axon physiology and regulation of neural circuit function. *Glia* 67 1–13. 10.1002/glia.23665 31233642 PMC6772175

[B272] Sun L. O. Mulinyawe S. B. Collins H. Y. Simon D. J. Tessier-Lavigne M. Correspondence B. A. B. (2018). Spatiotemporal control of CNS myelination by oligodendrocyte programmed cell death through the TFEB-PUMA axis. *Cell* 175 1811–1826. 10.1016/j.cell.2018.10.044 30503207 PMC6295215

[B273] Sun T. Hafler B. P. Kaing S. Kitada M. Ligon K. L. Widlund H. R. (2006). Evidence for motoneuron lineage-specific regulation of Olig2 in the vertebrate neural tube. *Dev. Biol.* 292 152–164. 10.1016/j.ydbio.2005.12.047 16469306

[B274] Swinburne I. A. Mosaliganti K. R. Green A. A. Megason S. G. (2015). Improved long-term imaging of embryos with genetically encoded α-bungarotoxin. *PLoS One* 10:e0134005. 10.1371/journal.pone.0134005 26244658 PMC4526548

[B275] Swire M. ffrench-Constant C. (2018). Seeing is believing: myelin dynamics in the adult CNS. *Neuron* 98 684–686. 10.1016/j.neuron.2018.05.005 29772200

[B276] Swire M. Kotelevtsev Y. Webb D. J. Lyons D. A. Ffrench-Constant C. (2019). Endothelin signalling mediates experience-dependent myelination in the CNS. *elife* 8 1–23. 10.7554/eLife.49493 31657718 PMC6831104

[B277] Takada N. Appel B. (2010). Identification of genes expressed by zebrafish oligodendrocytes using a differential microarray screen. *Dev. Dyn.* 239 2041–2047. 10.1002/dvdy.22338 20549738

[B278] Takada N. Kucenas S. Appel B. (2010). Sox10 is necessary for oligodendrocyte survival following axon wrapping. *Glia* 58 996–1006. 10.1002/GLIA.20981 20229602 PMC3639140

[B279] Tawk M. Makoukji J. Belle M. Fonte C. Trousson A. Hawkins T. (2011). Wnt/B-catenin signaling is an essential and direct driver of myelin gene expression and myelinogenesis. *J. Neurosci.* 31 3729–3742. 10.1523/JNEUROSCI.4270-10.2011 21389228 PMC6622795

[B280] Torvund-Jensen J. Steengaard J. Askebjerg L. B. Kjaer-Sorensen K. Laursen L. S. (2018). The 3’UTRs of myelin basic protein mrnas regulate transport, local translation and sensitivity to neuronal activity in zebrafish. *Front. Mol. Neurosci.* 11:185. 10.3389/fnmol.2018.00185 29946237 PMC6006989

[B281] Tsai H.-H. Niu J. Munji R. Davalos D. Chang J. Zhang H. (2016). Oligodendrocyte precursors migrate along vasculature in the developing nervous system. *Science* 351 379–384. 10.1126/SCIENCE.AAD3839 26798014 PMC5472053

[B282] Tsata V. Kroehne V. Wehner D. Rost F. Lange C. Hoppe C. (2020). Reactive oligodendrocyte progenitor cells (re-)myelinate the regenerating zebrafish spinal cord. *Devlopment* 147 1–16. 10.1242/dev.193946 33158923

[B283] Van Stegen B. Dagar S. Gottmann K. (2017). Release activity-dependent control of vesicle endocytosis by the synaptic adhesion molecule N-cadherin. *Sci. Rep.* 7 1–11. 10.1038/srep40865 28106089 PMC5247765

[B284] Vanwalleghem G. Schuster K. Taylor M. A. Favre-Bulle I. A. Scott E. K. (2020). Brain-wide mapping of water flow perception in zebrafish. *J. Neurosci.* 40 4130–4144. 10.1523/JNEUROSCI.0049-20.2020 32277044 PMC7244201

[B285] Villani A. Benjaminsen J. Moritz C. Henke K. Hartmann J. Norlin N. (2019). Clearance by microglia depends on packaging of phagosomes into a unique cellular compartment. *Dev. Cell* 49 77–88. 10.1016/j.devcel.2019.02.014 30880002

[B286] Vladimirov N. Mu Y. Kawashima T. Bennett D. V. Yang C. T. Looger L. L. (2014). Light-sheet functional imaging in fictively behaving zebrafish. *Nat. Methods* 11 883–884. 10.1038/nmeth.3040 25068735

[B287] Wake H. Lee P. R. Fields R. D. (2011). Control of local protein synthesis and initial events in myelination by action potentials. *Science* 333 1647–1651. 10.1126/SCIENCE.1206998 21817014 PMC3482340

[B288] Wan J. Goldman D. (2016). Retina regeneration in zebrafish. *Curr. Opin. Genet. Dev.* 40 41–47. 10.1016/j.gde.2016.05.009 27281280 PMC5135611

[B289] Wang Y. DelRosso N. V. Vaidyanathan T. V. Cahill M. K. Reitman M. E. Pittolo S. (2019). Accurate quantification of astrocyte and neurotransmitter fluorescence dynamics for single-cell and population-level physiology. *Nat. Neurosci.* 22 1936–1944. 10.1038/s41593-019-0492-2 31570865 PMC6858541

[B290] Warden M. R. Selimbeyoglu A. Mirzabekov J. J. Lo M. Thompson K. R. Kim S. Y. (2012). A prefrontal cortex-brainstem neuronal projection that controls response to behavioural challenge. *Nature* 492 428–432. 10.1038/nature11617 23160494 PMC5929119

[B291] Wheeler M. A. Jaronen M. Covacu R. Zandee S. E. J. Scalisi G. Rothhammer V. (2019). Environmental control of astrocyte pathogenic activities in CNS inflammation. *Cell* 176 581–596.e18. 10.1016/j.cell.2018.12.012 30661753 PMC6440749

[B292] White R. M. Sessa A. Burke C. Bowman T. LeBlanc J. Ceol C. (2008). Transparent adult zebrafish as a tool for in vivo transplantation analysis. *Cell Stem Cell* 2 183–189. 10.1016/j.stem.2007.11.002 18371439 PMC2292119

[B293] Widera D. Roger J. E. Thummel R. Iribarne M. Hyde D. R. Masai I. (2019). TNFα induces *Müller glia* to transition from non-proliferative gliosis to a regenerative response in mutant zebrafish presenting chronic photoreceptor degeneration. *Front. Cell Dev. Biol.* 7:296. 10.3389/fcell.2019.00296 31998714 PMC6962764

[B294] Williamson J. M. Lyons D. A. (2018). Myelin dynamics throughout life: an ever-changing landscape? *Front. Cell. Neurosci.* 12:424. 10.3389/fncel.2018.00424 30510502 PMC6252314

[B295] Wu S. Nguyen L. T. M. Pan H. Hassan S. Dai Y. Xu J. (2020). Two phenotypically and functionally distinct microglial populations in adult zebrafish. *Sci. Adv.* 6 21–25. 10.1126/sciadv.abd1160 33208372 PMC7673811

[B296] Wu S. Xue R. Hassan S. Nguyen T. M. L. Wang T. Pan H. (2018). Il34-Csf1r pathway regulates the migration and colonization of microglial precursors. *Dev. Cell* 46 552–563. 10.1016/j.devcel.2018.08.005 30205037

[B297] Xiao Y. Hoodless L. J. Petrucco L. Portugues R. Czopka T. (2021). Oligodendrocyte precursor cells sculpt the visual system by regulating axonal remodeling 2 3 chancellor’s building. *bioRxiv* [Preprint] 10.1101/2021.03.11.434829 PMC8904260 35241802

[B298] Xu J. Wang T. Wu Y. Jin W. Wen Z. (2016). Microglia colonization of developing zebrafish midbrain is promoted by apoptotic neuron and lysophosphatidylcholine. *Dev. Cell* 38 214–222. 10.1016/j.devcel.2016.06.018 27424497

[B299] Yang M. L. Shin J. Kearns C. A. Langworthy M. M. Snell H. Walker M. B. (2015). CNS myelination requires cytoplasmic dynein function. *Dev. Dyn.* 244 134–145. 10.1002/dvdy 25488883 PMC4368448

[B300] Yang R. Zhan M. Guo M. Yuan H. Wang Y. Zhang Y. (2021). Yolk sac-derived Pdcd11-positive cells modulate zebra fi sh microglia differentiation through the NF- κ B-Tgf β 1 pathway. *Cell Death Differ.* 28 170–183. 10.1038/s41418-020-0591-3 32709934 PMC7853042

[B301] Yang S. M. Michel K. Jokhi V. Nedivi E. Arlotta P. (2020). Neuron class–specific responses govern adaptive myelin remodeling in the neocortex. *Science* 370 1–10. 10.1126/SCIENCE.ABD2109 33335032 PMC7931669

[B302] Yergert K. M. Hines J. H. Appel B. (2019). Neuronal activity enhances mRNA localization to myelin sheaths during development. *bioRxiv* [Preprint]

[B303] Yildirim K. Petri J. Kottmeier R. Klämbt C. (2019). *Drosophila glia*?: few cell types and many conserved functions. *Glia* 67 5–26. 10.1002/glia.23459 30443934

[B304] Zaucker A. Mercurio S. Sternheim N. Talbot W. S. Marlow F. L. (2013). notch3 is essential for oligodendrocyte development and vascular integrity in zebrafish. *Dis. Model. Mech.* 6 1246–1259. 10.1242/DMM.012005 23720232 PMC3759344

[B305] Zeller N. K. Behar T. N. Dubois-Dalcq M. E. Lazzarini R. A. (1985). The timely expression of myelin basic protein gene in cultured rat brain oligodendrocytes is independent of continuous neuronal influences. *J. Neurosci.* 5 2955–2962. 10.1523/jneurosci.05-11-02955.1985 2414417 PMC6565180

[B306] Zhao X. He X. Han X. Yu Y. Ye F. Chen Y. (2010). MicroRNA-mediated control of oligodendrocyte differentiation. *Neuron* 65:612. 10.1016/J.NEURON.2010.02.018 20223198 PMC2855245

[B307] Zhou Q. Anderson D. J. (2002). The bHLH transcription factors OLIG2 and OLIG1 couple neuronal and glial subtype specification. *Cell* 109 61–73. 10.1016/S0092-8674(02)00677-3 11955447

[B308] Zuchero J. B. Barres B. A. (2015). Glia in mammalian development and disease. *Devlopment* 142 3805–3809. 10.1242/dev.129304 26577203 PMC4712885

[B309] Zuchero J. B. Fu M. Sloan S. A. Ibrahim A. Olson A. Zaremba A. (2015). CNS myelin wrapping is driven by actin disassembly. *Dev. Cell* 34 152–167. 10.1016/J.DEVCEL.2015.06.011 26166300 PMC4519368

