# Supplementary material for: Insights Into Central Nervous System Glial Cell Formation and Function From Zebrafish
Source: Front Cell Dev Biol. 2021 Nov 29;9:754606. doi: 10.3389/fcell.2021.754606 (PMC8666443; doi:10.3389/fcell.2021.754606)
Supplement: Supplementary file 1 [file Table_1.docx]

**Table 1**: Genetic and pharmacological manipulations to study glial cell formation and function in the zebrafish.

| **Biological process** | **Gene/ pathway/ Manipulation** | **Phenotype** | **Notes** | **Reference** |
| --- | --- | --- | --- | --- |
| Radial glial cell development/ function |  |  |  |  |
|  | *kif11* mutant and pharmacological inhibition | Mitotic arrest, accumulation of radial glia in M-phase of cell cycle |  | (Johnson et al., 2014) |
|  | *lcn2* (rat *Lcn2* expression) | Increased radial glia cellular process number and thickness |  | (Lee et al., 2009) |
|  | *hexb* mutant | Lysosomal abnormalities in radial glia | Lysosomal changes also in microglia | (Kuil et al., 2019a) |
|  | *eaat2b* MO and mutant | Enhanced neuronal excitation and abnormal swimming behaviours by mutant zebrafish | *slc1a2b* encodes Eaat2b | (McKeown et al., 2012) |
| Müller glial cell development/ function |  |  |  |  |
|  | *ale oko (ako)* mutant | Loss of Müller glial apical processes | The *ako* locus encodes part of the dynactin complex | (Jing and Malicki, 2009) |
|  | *aipl1b* mutant | Loss of timely regenerative response by Müller glia | *aipl1b* mutant, gold rush (gosh) | (Widera et al., 2019) |
|  |  |  |  |  |
|  | *tnfα* MO | Reduced Müller glia cell proliferation |  | (Nelson et al., 2013) |
|  | *ascl1a* MO | Reduced Müller glia cell proliferation during regeneration |  | (Fausett et al., 2008) |
|  | *sox2* MO  *sox2* overexpression | Reduced Müller glia cell proliferation during regeneration  Increased Müller glia cell proliferation |  | (Gorsuch et al., 2017) |
|  | *eaat2* MO | Reduced Müller glia electrographic response in larvae |  | (Niklaus et al., 2017) |
|  | *su(H)* MO | Lack Müller glia |  | (MacDonald et al., 2015) |
| Astrocyte development/ function |  |  |  |  |
|  | *fgfr3, fgfr4* | Reduced astrocyte cell volume and process elaboration | Cell type specific manipulation using CRISPR-CAS9 | (Chen et al., 2020) |
|  | Chemogenetic regulation of astrocyte activity | Increased passivity behaviour by zebrafish, but no effect to swim vigour |  | (Mu et al., 2019) |
| Oligodendrocyte lineage development |  |  |  |  |
|  | Notch pathway | Specification of OPCs in pMN domain | Various factors in pathway investigated | (Appel et al., 2001; Park and Appel, 2003; Ravanelli et al., 2018) |
|  | *boc* | Specification of OPCs in pMN domain and reduced oligodendrocyte number |  | (Kearns et al., 2021) |
|  | Hedgehog pathway | Specification of OPCs in pMN domain |  | (Park et al., 2004; Ravanelli et al., 2018) |
|  | *prdm8* | Controls the timing of the motor neuron to OPC production switch |  | (Scott et al., 2020) |
|  | Wnt pathway | Disrupted myelin gene expression, myelin compaction (CNS and PNS) and hypomyelination |  | (Tawk et al., 2011) |
|  | *olig2* MO | No OL lineage | Also reduced motor neuron lineage | (Park et al., 2002) |
|  | *olig1* overexpression | Promotes myelination | Needed to be co-expressed with *sox10* | (Li et al., 2007) |
|  | *nkx2.2a* MO | Prolongation of OPC proliferation and migration/ delay in differentiation | Effect on subset of OPCs | (Kucenas et al., 2008) |
|  | *fmr1* mutant | Reduced OL number | Also affects myelin sheath length | (Doll et al., 2020, 2021) |
|  | *notch3* mutants | Delay in OPC specification | CADASIL disease model | (Zaucker et al., 2013) |
|  | *gpr56* mutants | Reduced OPC proliferation |  | (Ackerman et al., 2015) |
|  | *met* mutant and pharmacological inhibitor | OPC migration | Preprint | (Ali et al., 2021) |
|  | *gria4a* mutant | OL migration | Separate role in myelination | (Piller et al., 2021) |
|  | VGCC pharmacological manipulation | Regulate OPC migration and myelination downstream of gria4a |  | (Piller et al., 2021) |
|  | *nf1a/b* MOs and mutants | Increased OPC proliferation and migration, but impaired myelination |  | (Lee et al., 2010; Shin et al., 2012) |
|  | *znf16l* mutant | Impaired OPC specification and migration, and reduced myelination | Phenotypes rescued by mouse zfp488 | (Sidik and Talbot, 2015) |
|  | *mir-219* MO | Impairs OL differentiation | Influencing progenitor polarity | (Zhao et al., 2010; Hudish et al., 2013) |
|  | *hmgcs1* mutant and pharmacology of pathway | Disrupts OPC migration to target axons and myelin gene expression |  | (Mathews et al., 2014) |
| Oligodendrocyte differentiation and myelination |  |  |  |  |
|  | *sox10* mutant | Death of pre-myelinating oligodendrocytes, no myelin |  | (Takada et al., 2010) |
|  | *tfeb* mutants | Precocious (excess) myelination |  | (Meireles et al., 2018) |
|  | *myrf* mutant | Hypomyelination |  | (Madden et al., 2021) |
|  | Ca2+ | Buffered Ca2+ reduces sheath growth |  | (Krasnow et al., 2018) |
|  | *calpastatin* expression in OLs | Prevents sheath retraction, so more sheaths per OL | Cell-type specific manipulation | (Baraban et al., 2018) |
|  | *neurofascin b* mutants | Myelin mistargeting  Impaired sheath growth | *neurofascin b*analogous to glial NFASC155 in mammals  Phenotypes confirmed in mice | (Djannatian et al., 2019; Klingseisen et al., 2019) |
|  | *caspr1* mutant | Impaired sheath growth | Conserved function in mammals | (Djannatian et al., 2019; Klingseisen et al., 2019) |
|  | *contactin1a* | Myelin mistargeting and impaired sheath growth | *contactin1a* and *1b* appear to have roles that collate as analogous to Contactin1 in mammals | (Djannatian et al., 2019) |
|  | Constitutively active *fyn* | More myelin sheaths per OL |  | (Czopka et al., 2013) |
|  | Dominant negative *fyn* | Fewer sheaths per OL |  | (Czopka et al., 2013) |
|  | Constitutive activation of *pak1* | Increased sheath number | Cell autonomous demonstration | (Brown et al., 2021) |
|  | Pak1 chemical inhibitor | Decrease sheath number per OL |  | (Brown et al., 2021) |
|  | *gria4a* mutant | Reduced myelination | Cell type specific role tested | (Piller et al., 2021) |
|  | Dominant negative *cadm1b* | More but shorter sheaths per OL | Cell-type specific manipulation | (Hughes and Appel, 2019) |
|  | Dominant negative *lrrtm* | More but shorter sheaths | Cell-type specific manipulation | (Hughes and Appel, 2019) |
|  | Dominant negative *nlg2b* | Fewer but longer sheaths | Cell type specific manipulation | (Hughes and Appel, 2019) |
|  | *ednrb* mutant  *pkce* agonist | Reduced sheath number  Rescues *ednrb* mutant sheath number | Conserved in rodents | (Swire et al., 2019) |
|  | *fbxw7* mutant and DN-*fbxw7* | Increased OPC specification  Increased myelination | DN-*fbxw7* cell type specific role in OLs  Mediated by *mtor* | (Snyder et al., 2012)  (Kearns et al., 2015) |
|  | *mtor* mutant or pharmacological blockade | Reduced myelination |  | (Kearns et al., 2015) |
|  | Constitutively active-*akt1* | Increased myelination, including inappropriate myelin targeting | CA-Akt1 cell type specific role in OLs | (Almeida et al., 2018) |
|  | Axonal-*ephrinA1* expression | Inhibition of myelination |  | (Harboe et al., 2018) |
|  | *ephA4* MO and inhibitor | Increased myelination |  | (Harboe et al., 2018) |
|  | *kif1b* mutant | No mbp mRNA transport to myelinating processes |  | (Lyons et al., 2009) |
|  | Dynein/ Dynactin mutants | Impaired oligodendrocyte production and myelin mRNA transport |  | (Yang et al., 2015; Herbert et al., 2017) |
|  | *slc12a2b* mutants | Buffering at periaxonal space and myelinated axon integrity | *slc12a2b* encodes NKCC1b | (Marshall-Phelps et al., 2020; Moyon et al., 2021) |
|  | Reduced vesicular release from all axons | Fewer sheaths made by OLs | Conserved in human | (Mensch et al., 2015; James et al., 2021) |
|  | Reduced vesicular release from single axons | Fewer and shorter sheaths |  | (Hines et al., 2015; Koudelka et al., 2016; Almeida et al., 2021) |
|  | Increased activity (all neurons) | More sheaths per OL  Increased *mbp* mRNA transport |  | (Mensch et al., 2015)  (Torvund-Jensen et al., 2018) |
|  | Increased activity (single neurons) | Longer sheaths per axon |  | (Hines et al., 2015; Koudelka et al., 2016; Almeida et al., 2021) |
| Microglial development/ function |  |  |  |  |
|  | *slc7a7* MO | Disrupted colonisation and microglial survival |  | (Rossi et al., 2015; Demy et al., 2021) |
|  | *hexb* mutant | Lysosomal abnormalities in microglia in early development and reduced locomotor activity in mutants from 5dpf | Lysosomal abnormalities also seen in radial glia | (Kuil et al., 2019a) |
|  | *gpr137b* mutant | Disrupted autophagy and increased lysosomal compartment in microglia | Gpr137b interacts with Rag GTPases to regulate mTORC1 translocation | (Gan et al., 2019) |
|  | *il34* mutant | Impaired microglial recruitment to the brain |  | (Wu et al., 2018; Kuil et al., 2019b) |
|  | *csf1r* mutants | Reduced microglia cell number, disrupted migration and CNS colonisation |  | (Oosterhof et al., 2018; Wu et al., 2018; Kuil et al., 2019b) |
|  | *pu.1* MO | Reduced myeloid cell development |  | (Lieschke et al., 2002; Rhodes et al., 2005) |
|  | *irf8* mutant | Lack microglia during early development |  | (Shiau et al., 2015) |
|  | *xpr1* mutants | Tissue resident macrophage differentiation inhibited, lack microglia |  | (Meireles et al., 2014) |
|  | *nlrc3*-like mutant | Primitive macrophage systemic inflammation, aggregation and failure to migrate to the brain |  | (Shiau et al., 2014) |
|  | *tnnt2a* MO | Reduced microglia cell number |  | (Xu et al., 2016) |
|  | *gpr132* MO | Reduced microglia cell number |  | (Xu et al., 2016) |
|  | *tim-4* MO | Impaired phagosome stabilisation in microglia |  | (Mazaheri et al., 2014) |
|  | *bai1* MO | Impaired phagosome formation and cargo transport in microglia |  | (Mazaheri et al., 2014) |
|  | *raga* mutant | Reduced microglia number and increased lysosomal compartments | Component of the Rag-Ragulator complex | (Shen et al., 2016) |
|  | *lamtor4* mutant | Reduced microglia number | Component of the Rag-Ragulator complex | (Shen et al., 2016) |
|  | *mTOR* mutant and pharmacological manipulation | Abnormal microglia cell morphology by 7dpf |  | (Shen et al., 2016) |
|  | *p2y12* MO | Reduced microglia cell number |  | (Sieger et al., 2012) |

dpf refers to days post fertilisation.
